# Evidence for somatic gene conversion and deletion in bipolar disorder, Crohn's disease, coronary artery disease, hypertension, rheumatoid arthritis, type-1 diabetes, and type-2 diabetes

**DOI:** 10.1186/1741-7015-9-12

**Published:** 2011-02-03

**Authors:** Kenneth Andrew Ross

**Affiliations:** 1 Department of Computer Science, Columbia University, New York, NY 10027, USA

## Abstract

**Background:**

During gene conversion, genetic information is transferred unidirectionally between highly homologous but non-allelic regions of DNA. While germ-line gene conversion has been implicated in the pathogenesis of some diseases, somatic gene conversion has remained technically difficult to investigate on a large scale.

**Methods:**

A novel analysis technique is proposed for detecting the signature of somatic gene conversion from SNP microarray data. The Wellcome Trust Case Control Consortium has gathered SNP microarray data for two control populations and cohorts for bipolar disorder (BD), cardiovascular disease (CAD), Crohn's disease (CD), hypertension (HT), rheumatoid arthritis (RA), type-1 diabetes (T1D) and type-2 diabetes (T2D). Using the new analysis technique, the seven disease cohorts are analyzed to identify cohort-specific SNPs at which conversion is predicted. The quality of the predictions is assessed by identifying known disease associations for genes in the homologous duplicons, and comparing the frequency of such associations with background rates.

**Results:**

Of 28 disease/locus pairs meeting stringent conditions, 22 show various degrees of disease association, compared with only 8 of 70 in a mock study designed to measure the background association rate (*P *< 10^-9^). Additional candidate genes are identified using less stringent filtering conditions. In some cases, somatic deletions appear likely. RA has a distinctive pattern of events relative to other diseases. Similarities in patterns are apparent between BD and HT.

**Conclusions:**

The associations derived represent the first evidence that somatic gene conversion could be a significant causative factor in each of the seven diseases. The specific genes provide potential insights about disease mechanisms, and are strong candidates for further study. Please see Commentary: http://www.biomedcentral.com/1741-7015/9/13/abstract.

## Background

Gene conversion is a process in which genetic information is transferred unidirectionally between highly homologous but non-allelic regions of DNA [1]. The genome contains many pairs of homologous regions, reflecting frequent gene duplication during evolution. Gene conversion is usually triggered by a double strand break (DSB), which can occur during meiosis or mitosis [1]. The DSB is repaired using the homologous sequence as the template. In mammalian cells, the sister chromatid is the most frequent conversion substrate [2], typically leading to perfect repair of a DSB. Gene conversion from other sequence, however, can lead to DNA changes. Gene conversion has recently been implicated in a number of diseases, as a source of both inherited and de-novo germ-line mutation [1]. It has been hypothesized that somatic gene conversion is relatively frequent but has escaped attention due to the technical difficulty of measurement [1].

**Figure 1:**
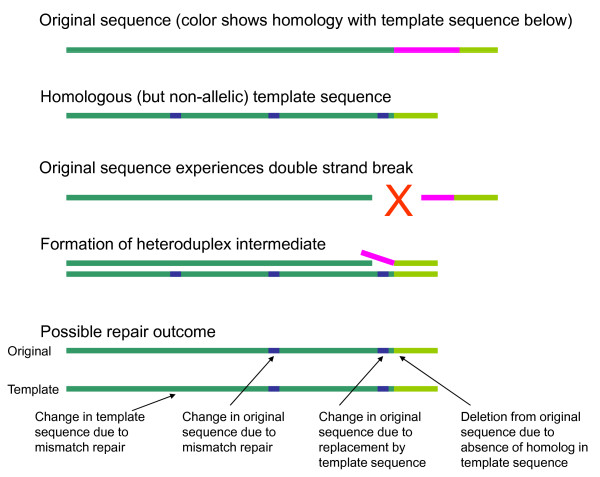
**A model of gene conversion between duplicons**. Two homologous but non-allelic sequences are shown, with homology indicated by a common green color. After a double strand break in the original sequence, the template sequence is used to form a heteroduplex DNA structure with the original sequence during the process of repair. A possible repair outcome is shown, illustrating changes to both the template and original sequences far from the location of the break, as well as changes and deletion in the original sequence in the vicinity of the break.

An informative example of gene conversion is the IDS gene, located on the X chromosome. Mutations in IDS cause Hunter syndrome. There is a pseudogene IDS2 located 20 kb from IDS in an inverted orientation relative to IDS, with 88% overall homology to IDS [3]. 20% of Hunter syndrome mutations involve structural rearrangements induced by the interaction of the two nonallelic homologous regions [3,4]. The rearrangements appear to be independent events, indicating a recurrent mutation rather than common ancestry. Observed rearrangements include deletions, inversions, and gene conversion events [3,4]. Among the regions exhibiting gene conversion, a complex pattern of alternating sequence fragments from each of the duplicons is apparent. The IDS2 pseudogene is missing several IDS exons, but exhibits homology with IDS on each side of this 'gap'. Some of the deletion events observed in the IDS gene appear to represent conversion of IDS sequence by IDS2 in the vicinity of this gap, leading to the elimination of those exons [4]. A one kilobase recombinational hotspot has been identified for the IDS/IDS2 events; this hotspot exhibits 98% identity compared with the 88% overall identity of the duplicons [3]. Lagerstedt *et al*. [3] suggest that recombination is initiated in this high-identity region, and spreads through branch migration until a region of sufficient sequence divergence is reached. Lagerstedt *et al*. propose a model in which gene conversion leads to changes in both duplicons, and in which mismatched base pairs in the heteroduplex DNA may be corrected to generate additional conversion [3]. Figure 1 illustrates this model. These observations lead to two important conclusions. First, when looking for evidence of gene conversion, one should examine *all *duplicons for a given sequence. Second, one should examine the entire contiguous high-homology sequence in those duplicons, and not limit the analysis to the immediate neighborhood of a particular locus.

Somatic gene conversion can have multiple kinds of effects. Most obviously, conversion of a coding sequence by a non-identical homologous sequence may lead to a dysfunctional gene product, or an immunogenic novel amino acid sequence. Conversion of a regulatory, promoter, suppressor, or enhancer sequence may alter gene expression, either up or down. Since converted sequence usually retains the methylation status of the source sequence [5], conversion may result in either the methylation of previously unmethylated promoter sequence, suppressing gene expression, or in the demethylation of previously methylated sequence, enabling gene expression where it was not previously expressed. Gene conversion may also be correlated with other effects of nonallelic homologous pairing. Crossover and conversion occur in the same hot spot regions, and gene conversion appears to be preferred over crossover when interacting regions are short [6]. Non-allelic crossover may lead to insertions, deletions and/or inversions. Homologous pairing within a short region of DNA could create DNA loop structures that alter transcription patterns [7]. Conversion could potentially occur during DNA/RNA pairing [8,9]. Any of the effects mentioned above could have a major impact on cell function, and provide plausible causative mechanisms for disease. 

I propose to examine somatic gene conversion in the context of disease using single nucleotide polymorphism (SNP) microarray data. Because conversion tracts are short, linkage disequilibrium (LD) between a gene conversion locus and nearby SNP markers is likely to be weak or nonexistent [10]. As a result, it becomes necessary to analyze single-SNP markers without expecting to see correlated patterns in nearby markers as one would expect in a traditional disease association study.

The Wellcome Trust Case Control Consortium (WTCCC) data set was obtained using an Affymetrix 500 K platform [11]. Genotyping was performed on two large British control populations (58C, NBS), in addition to disjoint populations for bipolar disorder (BD), Crohn's disease (CD), coronary artery disease (CAD), hypertension (HT), rheumatoid arthritis (RA), type-1 diabetes (T1D) and type-2 diabetes (T2D). The WTCCC data has been extensively analyzed using a traditional genomewide association study [11]. This previous analysis required the presence of three concordant SNP markers in order to identify a disease-associated haplotype. Such an analysis is likely to miss gene conversion events because of the weak LD. Further, by focusing the analysis at the called-genotype level, such an analysis is insensitive to somatic changes to the genome.

DNA samples in the WTCCC study are obtained from lymphocytes. One might be concerned that an analysis of somatic mutation in lymphocytes may not be informative about somatic mutation in other tissues more closely associated with the diseases in the WTCCC study. Fortunately, there is some evidence that a phenomenon related to gene conversion known as sister chromatid exchange (SCE) is informative about disease when measured in lymphocytes. SCE involves crossover between homologous sister chromatids mediated by the homologous recombination pathway [12], and has been interpreted as indicating general genome instability and/or a response to DNA damage [13]. SCE is elevated in lymphocytes of individuals with CD [14], CAD [15], T1D [16], and T2D [17], but not RA [18], although in some cases the elevation may be related to treatment rather than disease [19]. SCE is also elevated in multiple sclerosis [20], systemic lupus erythematosus [21], several cancers [14], and in individuals with viral infections [22].

Since SCE analysis using lymphocytes (rather than tissues directly affected by the disease) is informative, one might expect lymphocytes to also show disease-associated gene conversion behavior. Because blood cells are widely circulating, they are likely to encounter agents of double strand breakage such as viruses, and therefore exhibit gene conversion if conversion is occurring anywhere in the body. Further, a disease may be associated with damage to a particular tissue, for example by autoimmune processes, and the destroyed tissue is unavailable for analysis. Other cell types such as lymphocytes might therefore serve as useful proxies for damaged tissues. If the mechanisms of in-vivo gene conversion are sequence-specific rather than tissue-specific, then lymphocytes would exhibit the same conversion experienced by the damaged tissue, without eliciting the destruction response.

To identify somatic changes in a population I propose a novel data analysis technique. The technique takes advantage of the fact that a sample contains DNA from many cells of a single individual. If a significant proportion of those cells have undergone gene conversion at a locus, then the resulting change in the genotype of those cells should be measurable as a perturbation in the intensity for the two allele probes at that locus. An SNP with a distribution of perturbations specific to a disease population serves as a marker for a potential disease-associated locus. More details about how such perturbations are measured, and why such perturbations would have a signature different from other sources of variation such as paralogous sequence variants, can be found in the Methods section below.

Once a set of SNPs showing the signature of gene conversion is identified in a disease population, it would be desirable to validate those associations using an independent source of information that links the disease to those SNPs significantly more closely than to randomly chosen SNPs. As noted above, one needs to consider not just the SNP locus itself, but all regions with homology to the duplicon containing the SNP. The most direct form of association between a region and a disease is to find a gene in the region that is known to be associated with the disease, or that participates in a critical pathway known to be relevant for the disease. Additional evidence might include data showing that the gene is expressed in the relevant tissue with function related to disease pathogenesis. Most regions of high homology contain at most a few genes, and so the analysis can be relatively specific. One could also look for adjacent genes for which the duplicon could plausibly contain an upstream enhancer locus. I use 30 kb as a threshold for this type of adjacency.

When duplicons are nearby on the same chromosome, the intermediate region between them is an additional region of interest. Improper recombination between such regions could lead to inversions, insertions, or deletions of the intermediate sequence. In some situations, somatic deletion of a genomic region can generate patterns similar to those that would be generated by gene conversion. Deletion might be suspected when the duplicons occur in an aligned fashion nearby on the same chromosome, a configuration that could lead to misaligned recombination.

In the presence of an agent that induces genetic damage, a cell may respond by inducing the homology-directed repair pathway [23]. If this pathway is induced in each of many cells in response to the same agent, the same homology-biased mutations may happen in a variety of tissues. Mutations in stem cells will persist in lineages descending from those cells.

The damage-initiating agent may act locally or globally. A local agent, such as a virus that damages DNA in a position-specific manner, could induce gene conversion selectively in the region surrounding the target sequence. A global agent, such as a deficient or inactivated DNA repair pathway [24], would lead to DNA damage in a broad (but not necessarily random) fashion, inducing generalized gene conversion at many loci. Local gene conversion will be identifiable as a perturbation in the disease population that is absent in the control population and other disease populations. Perturbations due to global gene conversion may be present, to a lesser degree, in other populations whose diseases are caused by global agents. The perturbations should presumably be absent in the control population and in populations for diseases caused exclusively by local agents.

Increased SCE exchange rates are likely to be correlated with a global causative agent. Based on the SCE data for five of the seven studied diseases [14-18], one might hypothesize that RA is caused by a local agent, while CD, CAD, T1D, and T2D are caused by global agents. This hypothesis will be evaluated in the following analysis.

## Methods

Raw signal intensity and genotype calling data were obtained from the WTCCC in an anonymized form, and the analysis of the data was approved by a Columbia Institutional Review Board. Each disease cohort contained approximately 2,000 individuals, while the two control cohorts each contained approximately 1,500 individuals. The Affymetrix platform supports 500,568 SNP loci, of which 459,653 passed the WTCCC quality control procedures [11].

For a SNP locus with an A/B polymorphism, the microarray generates a pair of intensity values *I_A _*and *I_B_*. Each intensity value is the average intensity over a small number of oligonucleotide probes containing the allele together with some flanking sequence. The (*I_A_, I_B_*) point typically falls within one of three clusters corresponding to the three genotypes AA, AB, and BB.

**Figure 2:**
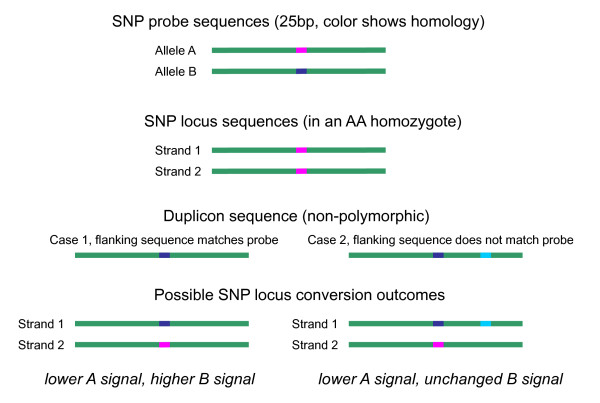
**Effects of gene conversion on probe intensity signals**. A microarray has two probes for a SNP, each 25 bp long (top). An individual with an AA homozygous genotype at the SNP locus is shown. Two examples of gene conversion are illustrated. The left example considers the case when the duplicon contains sequence that exactly matches the B probe. The right example considers the case when the duplicon contains sequence that does not match either probe.

Consider now an individual with an AA genotype. Suppose that 20% of the sampled cells of this individual have undergone gene conversion in which one of the A alleles has been converted into a B allele by a homologous sequence, while the flanking sequence has remained unchanged. The left example of Figure 2 shows this kind of conversion. (Conversion of both A alleles would be rare, and is ignored.) This individual will display an overall (*I_A _, I_B_*) intensity pair that is 20% of the way from the AA cluster to the AB cluster. In another individual with a heterozygous AB genotype, a 20% conversion rate at the same locus would yield an overall (*I_A _, I_B_*) intensity pair that is 10% of the way from the AB cluster to the BB cluster, since only the conversion of the A allele will cause a change in probe intensities. In an individual with a BB genotype, no change would be observed.

**Figure 3:**
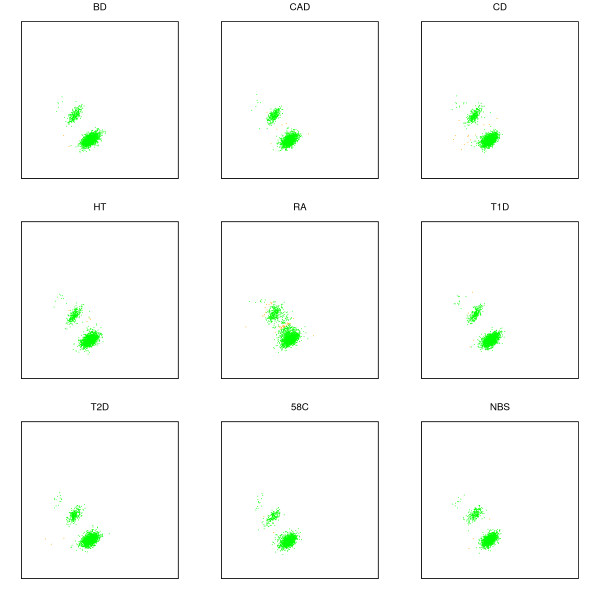
**Cluster plots for SNP rs4988327 in the WTCCC data**. Note the high spread for RA, and the resulting increase in no-calls (orange) relative to calls (green).

Because there is experimental variation in intensity measurements, it may be difficult to determine whether a small perturbation in a single measurement represents gene conversion or merely noise. However, it is possible to study the distribution of perturbations for a population at a locus. If a population has a significant spread of intensities between clusters, when control populations do not, then one can hypothesize that gene conversion at that locus is happening in a population-specific manner. See the cluster plot for RA in Figure 3 for an example. If the population is a disease cohort, then the locus may be associated with the disease phenotype.

Returning to the example above, consider the complementary situation in which the flanking sequence near an SNP probe has been converted. Whether or not the SNP locus is changed, the converted sequence will no longer match either probe sequence. The right example of Figure 2 shows this kind of conversion. If many cells in an individual are converted in this fashion, a reduced signal from this sequence will be measured by both probes of the microarray. For a locus at which this effect is associated with the disease phenotype, all clusters will shift radially towards the origin in the cluster plots for the disease population. 

Calling algorithms attempt to identify the boundaries of clusters corresponding to the AA, AB and BB genotypes. For example, the Chiamo algorithm [11], considers all populations simultaneously, and estimates cluster boundaries in a way that allows for some population-dependent differences. The intensity distributions vary from SNP to SNP, and so clustering is performed separately for each SNP.

Based on the analysis above, gene conversion for a particular population should be accompanied by either (a) an increase in the spread of the two-dimensional intensity distribution relative to the control population, or (b) a translation of the clusters towards the origin, relative to the control population. In case (a), there should be an increase in the number of points that are either between clusters, or on the fringe of a cluster. In case (b), there should be a decrease in the distance between clusters, leading to an increase in the number of points whose cluster assignment is ambiguous. Either way, there will be an increase in the number of no-calls generated by the calling algorithm, relative to the control populations. This is one 'signature' of gene conversion that I will try to identify.

The Chiamo calling algorithm has been applied to the WTCCC data, and it is possible to use those calls to help recognize the signature of gene conversion. Chiamo generates a confidence score for a call; the authors of the Chiamo algorithm recommend that when this score is below 0.9, the genotype should be considered a 'no-call.' When clusters are more dispersed, their peripheries can begin to overlap with each other. In such a situation, the Chiamo algorithm will have less certainty about points falling in the intermediate regions. Chiamo will define cluster boundaries more tightly, resulting in an increase in the no-call rate for intermediate points [11]. An example of this phenomenon is given in Figure 3, where the orange points (that are particularly frequent in RA at this locus) are no-calls.

An increase in no-calls between two clusters can lead to a biased allele distribution in the called genotypes. For example, if there are many no-calls between the AA and AB clusters, then the A allele will be underrepresented among the subpopulation whose genotypes are called with high confidence. This bias is another possible signature for gene conversion. (See Additional file 1 for an extended discussion of no-calls.) Note that there may be cases of gene conversion that do not show this signature because the non-called points do not change the observed allele frequencies.

To identify gene conversion events, I take three complementary approaches. The first approach that I call the 'stringent' filter is designed to optimize precision, that is, to minimize the number of false positives while possibly missing some true positives. The second approach is designed to provide better recall, that is, to include more true positives at the risk of also including false positives. This second approach is called the 'relaxed' filter. The third approach, termed the 'no-call-only' filter, looks only for extreme no-call rates, since some gene conversion loci may not exhibit changes in called allele frequencies.

For the stringent filter, called SNPs with high no-call rates in a population relative to the union of the two control populations are initially selected. A chi-squared statistic is calculated for each SNP based on a 2 × 2 chi-squared test comparing calls/no-calls for both the disease population and the control population. Only SNPs with an increase in the no-call rate in the disease population and a chi-squared statistic corresponding to *P *< 5 × 10^-5 ^in a one-sided test are retained by this initial selection.

A further selection is applied to test for a bias in the genotype distribution in the disease population relative to controls. Bias is assessed in one of two ways; an SNP that displays bias according to either of these tests is retained. Only SNPs in which the control population has at least ten individuals for each of the AA, AB and BB genotypes are considered. First, the three genotype frequencies in the disease population are compared with the corresponding frequencies in the control population using a 3 × 2 chi-squared test to determine the likelihood that they have a common distribution. Only SNPs with a chi-squared statistic corresponding to *P *< 5 × 10^-4 ^in a two-sided test are retained. Second, the three genotype frequencies in the disease population and control population are separately assessed for departure from Hardy-Weinberg Equilibrium using a conventional 3 × 2 chi-squared test. Only SNPs with a chi-squared statistic corresponding to *P *< 5 × 10^-4 ^in a two-sided test in the disease population and a chi-squared statistic corresponding to *P *> 0.01 in the control population are retained.

Gene conversion appears to require at least 300 base pairs of homology in humans [1]. Among known gene conversion loci, the smallest degree of identity between the homologous regions is 88% [1]. One should therefore not expect newly discovered loci to have identity much below 88%. I will thus use 85% identity as a lower bound for the stringent filter.

The candidate SNPs were evaluated for homologous flanking sequence elsewhere in the genome. The UCSC database of segmental duplications [25] was used to identify genomewide duplications with at least 1,000 base pairs of homology (after elimination of low-complexity repeats) and at least 90% identity. Additionally, each SNP that met the other stringent filter conditions was subjected to manual analysis using the BLAST network service at NCBI to identify duplications that may not meet the thresholds of the segmental duplication database, but that may still be relevant for gene conversion. (I used the Megablast algorithm with default parameters. When a duplicon contains several almost-contiguous segments, the identity of the duplicon is the identity reported by BLAST for the segment containing the region that maps to the SNP under consideration.) The three filters are summarized in Table 1. The relaxed and no-call filters use different homology criteria from the stringent test so that the segmental duplication database can be used to automate the analysis. Because the segmental duplication database excludes regions with low complexity repeats, some SNPs in regions with more than 90% homology (for example, rs9378249) are not in the segmental duplication database.

**Table 1:** Summary of the three data filters.

Filter	No-call rate increase	Min. homology	Biased distribution
Stringent	*P *< 5 × 10^-5^	300 bp, 85%	*P *< 5 × 10^-4^
Relaxed	*P *< 5 × 10^-2^	1,000 bp, 90%	*P *< 1 × 10^-2^
No-call only	*P *< 1 × 10^-8^	1,000 bp, 90%	-

The analysis does not consider SNPs on the Y chromosome. For the X chromosome, the analysis is limited to the female subpopulation within each cohort. As a result, some statistical power is lost, particularly for cohorts such as CAD that have a relatively small number of female members.

Cluster plots for all SNPs mentioned in the text can be found in Additional file 2.

### Sources of variation

Copy number variations at an SNP locus mean that in addition to the conventional AA, AB, and BB genotypes, there may be additional genotypes such as AAB and B. Each of these alternative genotypes would have its own cluster in the cluster plot, which can be examined for signs of more than three clusters. Each SNP was also assessed for known copy-number variation using the Database of Genomic Variants [26], since copy-number variants could also cause changes in no-call frequencies and genotype distributions that may be related to disease. (See Additional file 1 for further discussion of copy number variation.) Note that somatic deletion would generate genotypes like B in some cells, but since most cells retain the normal copy number, the effect will be a small perturbation in the cluster plot rather than a separate cluster. Germ-line mutations would not give the same perturbation patterns as somatic conversion. For a germ-line mutation that changed one allele to another, the individual would appear as part of another cluster in the corresponding cluster plot. If a germ-line mutation deleted or duplicated an allele, then the individual would appear as part of a cluster with a nonstandard copy number. If this deletion/duplication was common, then the cluster plot would show features typical of CNV loci, such as the presence of more than three clusters.

A paralogous sequence variant occurs when the homologous sequence to the mapped SNP sequence possesses a polymorphism. Suppose an SNP has probes for alleles A and B. If the paralogous sequence also has an A/B polymorphism, then the cluster plot will have five clusters, corresponding to AAAA, AAAB, AABB, ABBB, and BBBB. If the paralogous sequence has an A/C polymorphism, then the probes will not detect the signal from the C allele, and there will be clusters for AA, AB, BB, AAA, AAB, ABB, AAAA, AAAB, AABB. In either case, the cluster plot will differ significantly from what is expected under a gene conversion hypothesis.

Some polymorphisms on the microarray platform may have been misidentified, with the true polymorphism being in paralogous sequence with no polymorphism at the mapped SNP locus. As long as the paralogous sequence is part of a larger region of homology with the mapped SNP locus, the outcome of the gene conversion analysis will be unchanged by such phenomena because both duplicons are examined. 

A foundational somatic mutation could occur during early development, leading to a lineage of cells within the individual carrying the mutation. This kind of mutation will not be identified by the present analysis unless the blood cells being genotyped come from more than one such lineage. Even then, the relevance of a foundational mutation to disease would be unclear because the mutation would also have to have been in a lineage ancestral to the diseased tissue.

## Results

### Putative gene conversion events detected using the stringent filter

31 instances of putative gene conversion with duplicon identity of at least 85% were identified using the stringent filter, covering 23 distinct SNPs. This data is summarized in Tables 2, 3 and 4; additional information about the associations can be found in Table S1 in Additional file 1. The SNPs in Table 4 fall within the MHC region and are identified by the stringent filter for T1D. Since T1D has significant associations at the haplotype level in the MHC region [11], it is difficult to separate a conversion signal from the broader association signal for these SNPs. The same is true for RA [11], but no MHC SNPs were identified for RA using the stringent filter.

**Table 2:** SNPs identified for various cohorts using the stringent filter (Part 1).

Cohort(s)	SNP/identity (degeneracy)	Chr: Pos. (hg17) and orientation		Duplicon length	Characterized genes and pseudogenes in duplicons
CD	rs4471699	16:	30.2 M→	147 kb	SULT1A3, GIYD2, BOLA2, IMAA, CORO1A
	99.6%	16:	29.4 M→	146 kb	SULT1A3, GIYD2, BOLA2, *IMAA*, MLAS
	98.1%	16:	21.8 M→	41 kb	[UQCRC2]
	98.0%	16:	22.3 M←	42 kb	[NPIPL3]
	98.0%	16:	21.3 M→	42 kb	*IMAA*, [NPIPL3]
	97.1%	16:	18.8 M→	75 kb	*SMG1*
					
RA	rs669980	9:	0.2 M→	193 kb	CBWD1, FOXD4, FAM138A, WASH1, [DOCK8]
	98.9% (F)	2:	114 M←	189 kb	CBWD2, FOX4DL1, FAM138B, WASH2P, [RABL2A]
					
CAD, T2D	rs10502407	18:	10.6 M→	52 kb	-
	97.9%	18:	12.2 M←	64 kb	[CIDEA]
					
CAD	rs12134625	1:	78 M→	931	-
	97.0%	1:	24 M←	932	*FUSIP1*
					
BD, CAD	rs9551988	13:	19.2 M→	2.6 kb	*PSPC1*
HT	96.2% (F)	13:	18.7 M→	2.8 kb	[TUBA3C]
					
HT	rs935019	2:	127,162 K→	3.6 kb	*GYPC*
	95.3% (F)	2:	127,166 K→	3.5 kb	*GYPC*
					
HT	rs12227938	12:	37 M→	154 kb	ALG10B
	95.3% (P)	12:	34 M→	127 kb	ALG10
					
T2D	SNP_A-1797773	16:	45 M→	14 kb	*VPS35*, [ORC6L]
	94.8% (F)	16:	34 M←	16 kb	-
					
T1D	rs12381130	16:	5 M→	88 kb	ALG1, FAM86A
	94.7%	3:	127 M←	76 kb	ALG1L
	94.6%	11:	67 M→	79 kb	-
	94.6%	11:	71 M←	40 kb	FAM86C, [DEFB108B]
	94.5%	11:	3 M→	91 kb	[ZNF195]
	94.3%	3:	76 M→	44 kb	[FAM86D]
	94.0%	4:	9 M←	120 kb	-
	93.9%	3:	131 M→	44 kb	-
	93.9%	4:	4 M→	53 kb	-
	93.7%	12:	8 M→	53 kb	[FAM90A1]
	93.6%	8:	12 M→	41 kb	[FAM86B1]
	93.5%	8:	8 M←	63 kb	-

Multiple almost-contiguous segmental duplications are treated as a single large duplicon (intervening sequence is included in the length). The table includes only duplicons with at least 85% identity to the region containing the SNP. Duplicons with identical flanking sequence to the SNP are labeled as fully degenerate (F); duplicons with partial degeneracy are labeled (P). Characterized genes are listed if they occur within a duplicon. Genes in square brackets are outside the duplicon, but the duplicon is at most 30 kb upstream of the gene. Genes for a SNP are italicized if the SNP is within that gene, or if the SNP maps to a position within that gene in the duplicon.

**Table 3:** SNPs identified for various cohorts using the stringent filter (Part 2).

Cohort(s)	SNP/identity (degeneracy)	Chr: Pos. (hg17) and orientation		Duplicon length	Characterized genes and pseudogenes in duplicons
CD	rs11060028	12:	128 M→	1.5 kb	*GLT1D1*
	93.4% (P)	10:	102 M←	1.2 kb	[ABCC2]
					
T1D	rs3805006	3:	4,775 K→	402	*ITPR1*, [EGO]
	93.4% (P)	3:	4,773 K←	407	*ITPR1*, [EGO]
					
BD, HT	rs9378249	6:	31.4 M→	27 kb	HLA-B, DHFRP2
	92.9% (F)	6:	31.3 M→	35 kb	HLA-C
					
HT	rs841245	12:	27.1 M→	84 kb	-
	92.0% (P)	12:	27.6 M→	82 kb	*PPFIBP1*
					
BD	rs12070036	1:	224 M→	9 kb	*ZNF678*
	91.9%	12:	7 M←	3.5 kb	[PEX5]
	91.1%	12:	123 M←	10 kb	[RILPL1], [TMED2]
	90.9%	11:	26 M←	2.7 kb	-
					
RA	rs4988327	11:	68 M→	104 kb	LRP5
	91.2%	22:	24 M←	64 kb	LRP5L
					
T2D	rs11010908	10:	37.2 M→	6 kb	-
	90.6%	10:	27.2 M←	12 kb	-
	90.0%	10:	27.6 M→	6 kb	-
					
CAD	rs295470	3:	141 M→	1.9 kb	*ACTGP1*, [RBP2]
	89.5%	17:	77 M←	2.3 kb	*ACTG1*, [FSCN2]
	89.1%	1:	92 M←	866	-
	87.8%	X:	53 M←	636	-
	86.6%	2:	108 M→	568	-
	86.5%	17:	17 M→	696	[FLCN]
	85.9%	3:	12 M→	1.9 kb	*SYN2*
					
BD, HT	rs2122231	3:	35 M→	4.9 kb	-
	88.8%	6:	117.0 M→	4 kb	[NT5DC1]
	88.6%	18:	5 M→	3.9 kb	-
	88.5%	2:	194 M→	1 kb	-
	87.9%	1:	96 M→	4.9 kb	-
	87.3%	10:	117 M→	4.2 kb	-
	86.3%	20:	24 M→	728	-
					
BD, HT	SNP_A-1948953	17:	17 M→	894	*LNX1 pseudogene LOC644909*
	87.0% (P)	4:	54 M←	21 kb	*LNX1*
					
CD	rs9839841	3:	16 M→	110 kb	*RFTN1*
	86.8% (F)	Y:	7.6 M→	100 kb	*RFTN1-pseudogene LOC360015*, [TTTY12], [TTTY16]
					
BD, T2D	rs4850057	2:	4 M→	4.7 kb	-
	86.8%	9:	35 M→	4.5 kb	*UNC13B*
	86.1%	11:	5 M→	3.0 kb	[TRIM68], [OR51D1], [OR51E1]

**Table 4:** SNPs identified in the MHC region for T1D using the stringent filter.

Cohort(s)	SNP/identity (degeneracy)	Chr: Pos. (hg17) and orientation		Duplicon length	Characterized genes and pseudogenes in duplicons
T1D	rs9378249	6:	31.4 M→	27 kb	HLA-B, DHFRP2
	92.9% (F)	6:	31.3 M→	35 kb	HLA-C
					
T1D	rs9257223	6:	29 M→	16 kb	-
	92.5%	11:	50 M→	16 kb	-
					
T1D	rs389600	6:	30 M→	4 kb	HLA-K
	87.5%	6:	30 M←	4 kb	HLA-A
	87.5%	6:	30 M←	4 kb	HLA-H
	86.2%	6:	30 M←	4 kb	HLA-J
	85.8%	6:	30 M←	3.5 kb	HLA-G

In all 28 of the 28 instances in Tables 2 and 3, the change in allele frequency is consistent with what would be predicted by a gene conversion hypothesis (see Additional file 1). Additional SNPs that met the stringent filter conditions except that identity between duplicons was 71%-83% are discussed in Additional file 1.

The strength of the evidence for a putative SNP/disease association is determined by consulting the published literature in search of a known association. The strength of the evidence is summarized using the scale of Table 5, where a higher number corresponds roughly to stronger evidence. The score for a SNP is the maximum score for any gene in any duplicon associated with the SNP; genes for which a duplicon occurs 30 kb or less upstream of the gene are included. Note that the score for an SNP does not give any weight to genes occurring between neighboring homologous regions (except for the 30 kb-upstream genes mentioned above). The evidence score therefore ignores the possible deletion and/or duplication of genes in the intervening sequence. The code for the strength of the evidence is given in parentheses in the heading for each SNP.

**Table 5:** Numeric codes describing the strength of evidence for an association of a gene with a disease.

Code	Kind of evidence
6	Known association of the gene with the disease.
5	Gene is known to interact with an intermediate, and the intermediate has a known association with the disease.
4	Known association of the gene with a function central to disease pathogenesis (for example, insulin secretion for diabetes).
3	Gene is known to interact with an intermediate, and the intermediate has a known association with a function central to disease pathogenesis.
2	Known association of a region containing the gene with the disease.
1	Gene disruption is known to have a general mutagenic effect.
0	No evidence.

To assess the significance of the set of identified regions, the duplicons for the SNPs identified by the stringent test (which should have few false positives) are assessed for association with the corresponding disease. The code for the strength of the evidence is given in parentheses in the heading.

#### rs4471699 in CD (6)

Of the characterized genes in the various duplicons, SULT1A3 has the most obvious connection to the CD phenotype involving inflammation of the small and/or large intestine. SULT1A3 is highly expressed in the small intestine [27] where it specifically sulfates dopamine and is important for the metabolism of several neurotransmitters [28]. SULT1A3 shows reduced expression in the colons of CD patients [29]. (The related genes SULT1A1 and SULT1A2, which are also located in a segmentally duplicated region of chromosome 16, have reduced expression in CD [30].) Eisenhofer *et al*. [28] suggest that the production of dopamine sulfate in the intestine 'reflects an enzymatic "gut-blood" barrier for detoxifying dietary biogenic amines.' Dysfunction of this pathway could lead to toxicity in the small and large intestines.

The UQCRC2 gene is a part of the mitochondrial respiratory complex III. Apolipoprotein E4 binds to UQCRC2, and overexpression of a fragment of this protein impairs the function of complex III [31]. Mitochondrial dysfunction has been associated with CD in several case reports, including one with dysfunction in complex III [32].

Strikingly, the duplicon containing rs4471699 and the closest matching duplicon have recently been shown to be endpoints of a region deleted in the germ-line in certain cases of autism, and duplicated in others [33,34]. Among the common features of autism are gastrointestinal abnormalities [35]. Mitochondrial dysfunction also occurs with increased frequency in autism [36,37].

#### rs669980 in RA (5)

CBWD1 (and by inference also CBWD2) has 25% protein identity with the cobW gene of P. dentrificans that is thought to be involved in vitamin B_12 _processing [38], and possibly cobalt chelation [39,40]. Vitamin B_12_-binding proteins are found in the synovium of RA patients [41,42]. Low serum vitamin B_12 _levels are noted in a significant percentage of RA patients [43]. Methyl B_12 _appears to suppress cytokine production in T lymphocytes [44], which may be relevant to RA. Improper vitamin B_12 _processing can lead to elevated plasma homocysteine levels, which has been observed in multiple RA cohorts [45].

Dysregulation of cobalt chelation could also have secondary mutagenic effects, since cobalt is genotoxic [46].

#### rs10502407 in T2D (6), CAD (6)

CIDEA has known associations to obesity, insulin resistance, and T2D [47-49], which are also risk factors for CAD [50]. The duplicon is located upstream of CIDEA in a potential enhancer locus.

#### rs12134625 in CAD (3)

The FUSIP1 gene specifically represses splicing during mitosis [51,52] and in cells subject to heat shock [53]. Cells lacking FUSIP1 are defective in recovery after heat shock [53]. Splice repression after heat shock prevents the possible accumulation of inaccurately spliced mRNAs, until the heat-damaged splicing apparatus is restored to normal [53]. FUSIP1-null mice display multiple cardiac defects during embryonic development, due to improper processing of pre-mRNA encoding cardiac triadin [54]. Somatic defects in FUSIP1 that lead to mis-spliced triadin transcripts could be a pathogenic mechanism in CAD.

#### rs9551988 in CAD (3), BD (3), HT (3)

PSPC1 has sequence-specific RNA-binding domains, and localizes to paraspeckles [55]. While the function of paraspeckles is not fully understood, Prasanth *et al*. [56] describe how paraspeckles store CTN-RNA, which is cleaved under conditions of stress and released for immediate translation into protein. Prasanth *et al*. argue that this mechanism allows the cell to provide a rapid stress response, rather than having to wait for RNA transcription [56]. The released mRNA encodes SLC7A2, also known as CAT2, a cationic amino acid transporter involved in L-arginine transport, a necessary step in nitric oxide (NO) synthesis [56,57]. Insulin directly effects vascular endothelium and smooth muscle via nitric oxide release [58,59]. The pathway for insulin-induced NO synthesis involves L-arginine transport and the SLC7A2 gene [58,60,61]. The physiological implications of a dysregulation of insulin in obesity, CAD, and HT are well known [58,59]. A dysregulation of SLC7A2 function could have similar effects. In preeclampsia (HT and proteinuria in pregnancy) the L-arginine NO system of circulating leukocytes appears dysregulated [62]. The L-arginine NO pathway appears to be involved in the pathogenesis of BD [63,64].

#### rs935019 in HT (4)

The two duplicons are immediately adjacent and aligned within the GYPC gene. Such an arrangement provides an opportunity for improper recombination due to misalignment. Indeed, deletion variants of the GYPC gene have been attributed to unequal crossover at these duplicons [65]. One of these deletions frequently occurs spontaneously in E. coli during cloning [65], suggesting that spontaneous somatic deletions are also likely.

The GYPC gene codes for the GPC and GPD proteins, which regulate the shape and mechanical properties of red blood cells [66]. While there is no direct evidence linking GYPC to HT, the tissue-specificity and function of GYPC make such a link plausible.

#### rs12227938 in HT (3)

The HERG gene encodes pore-forming alpha-subunit protein important for repolarizing K^+ ^current in the heart [67]. The ALG10B gene (also known as KCR1) modulates HERG, reducing the sensitivity of cardiac cells to arrhythmic disturbance [68,69]. ALG10B suppresses heart rhythm and regulates cardiac automaticity [70]. Polymorphisms on ALG10B are associated with the risk of acquired long QT syndrome, a cardiac rhythm disturbance [71]. Somatic defects in ALG10B would have direct relevance to HT.

#### SNP A-1797773 in T2D (4)

VPS35 is part of the retromer protein complex, which has a variety of sorting-related functions [72]. Mutant VPS35 is associated with improper insulin secretion [73].

#### rs12381130 in T1D (0)

This particular duplicon has homology with many other regions. Interestingly, on chromosomes 3, 4, 8, and 11 there are pairs of homologous duplicons about 4 Mb apart. Gene conversion at rs12381130 could be a marker of more general conversion and/or improper recombination at these locations, potentially leading to somatic deletions, duplications or inversions of the sequence between duplicon pairs on a chromosome.

#### rs11060028 in CD (6)

GLT1D1 appears to be a glycosyltransferase, but relatively little is known about its specific function. The chromosome 10 duplicon is 16 kb upstream of ABCC2 in a possible enhancer locus. ABCC2 is expressed on the apical membrane in the jejunum, ileum and colon [74]. It is an efflux transporter, responsible for extruding toxic substances from the cell [29,74]. ABCC2 expression is reduced in CD, in both the ileum and colon [29].

#### rs3805006 in T1D (4)

rs3805006 is located within an intron of ITPR1, and 7 kb upstream of the noncoding RNA gene EGO [75]. ITPR1, together with the related receptors ITPR2 and ITPR3, regulate calcium release within the insulin secretion pathway in pancreatic beta cells [76]. The ITPR3 gene was associated with T1D in a Swedish population [77], although see [78,79].

#### rs9378249 in BD (0) and HT (0)

This SNP falls within the MHC region on chromosome 6. There is no general association of the MHC region with either BD or HT in the WTCCC data [11], although the region has recently been implicated in schizophrenia [80].

In the cluster plots for rs9378249, the no-calls for BD, HT, and T1D are located in the middle of the heterozygote cluster. This kind of clustering pattern strongly suggests variation between populations in the magnitude of the intensity measurements. Intensity variations could be a result of either somatic gene conversion or somatic deletion in certain populations, assuming in both cases that the control populations have higher intensity than the affected populations.

**Figure 4:**
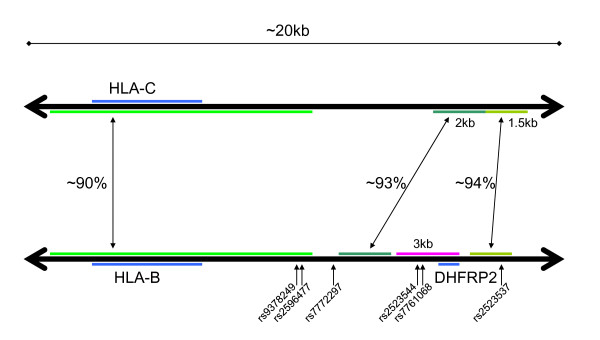
**The structure of the homology between the HLA-B and HLA-C containing duplicons on chromosome 6**. Genes and pseudogenes are shown in blue. Corresponding homologous regions are shown in matching shades of green, together with the degree of homology according to the segmental duplication track of the UCSC browser (the two rightmost segments) or Blast (the leftmost segment). The pink region is about 91% homologous to the DHFR region on chromosome 5.

A diagram of the homology between the two duplicons is given in Figure 4. From this diagram, it becomes apparent that conversion of the lower region by the upper region could eliminate the DHFRP2 sequence entirely.

Relatively low raw intensity levels at a locus would be expected if there were a significant number of deletions at that locus in somatic cells. Low intensity at rs7761068, which resides in the putatively deleted region and is the closest SNP to DHFRP2 in the microarray data set, could be interpreted as an indicator of more frequent somatic deletion of the region containing DHFRP2.

To determine a threshold for low/high intensity at rs7761068, the two control populations were pooled and the three genotype clusters were analyzed separately. For the first homozygous cluster, which is close to the y-axis in the cluster plot, the median y-intensity is 1.227. For the second homozygous cluster, which is close to the x-axis in the cluster plot, the median x-intensity is 1.493. For the heterozygous cluster, the median (x + y)-intensity is 1.888. Based on these numbers, an individual is defined to have low intensity at rs7761068 if the x, y, and x + y values are all lower than the corresponding thresholds; otherwise the individual is said to have high intensity at rs7761068. Each of the populations was then partitioned into low and high intensity fractions.

**Figure 5:**
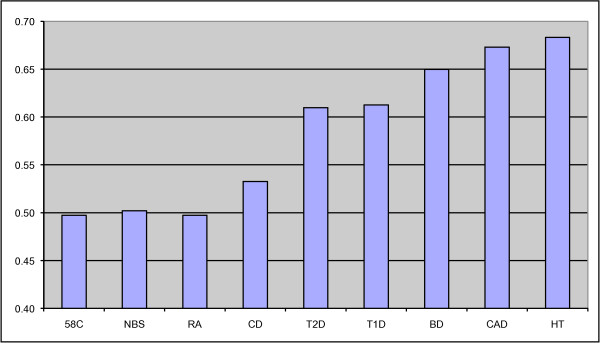
**Proportion of each of the nine populations having low measured intensity at rs7761068**. The intensity thresholds were chosen so that 50% of the combined control population would have low intensity.

The results shown in Figure 5 strongly suggest that there is increased deletion in all disease populations besides RA. (A 2 × 2 chi-squared test comparing each population with the combined controls yields *P *= 0.02 for CD, and *P *< 10^-13 ^for the other five populations.) rs9378249 displays an intensity distribution with features similar to rs7761068 shown in Figure 5, suggesting that deletion due to conversion and/or deletion of the green regions is more likely to be responsible than interactions between the pink region containing DHFRP2 and the region containing DHFR.

DHFRP2 is a pseudogene with homology to DHFR. DHFR codes for dihydrofolate reductase, an enzyme required for the synthesis of thymine nucleotides. Impaired T synthesis causes misincorporation of uracil into DNA, leading to various kinds of DNA damage [81]. While DHFRP2 is noncoding, its mRNA could interact with DHFR mRNA via an antisense regulatory mechanism [82]. The DHFR gene locus shows evidence of both sense and antisense transcription [83], consistent with a role for antisense regulation. (Since interactions of DHFRP2 and DHFR have not been demonstrated, the evidence level of this hypothesis is zero.)

In BD patients, folate sensitive fragile sites are expressed more often than in controls [84]. Polymorphisms in the MTHFD1 gene, which encodes several folate enzymes, are associated with BD [85]. Polymorphisms in the MTHFR gene, which encodes 5,10-methylenetetrahydrofolate reductase, have been associated with HT [86] and BD [87]. High homocysteine levels, which are often associated with folate deficiency, are associated with hypertension [88], and folate supplementation appears to decrease the risk of developing HT [89].

#### rs841245 in HT (5)

PPFIBP1 encodes the liprin-beta-1 gene, which is highly expressed in the heart [90]. Liprin-beta-1 interacts specifically with the S100A4/Mts1 protein *in vivo *[91]. The S100A4/Mts1 protein is more highly expressed in individuals with HT, and appears to cause changes in vasculature [92-95].

#### rs12070036 in BD (5)

ZNF678 has unknown function. It has diverged significantly from all other known zinc-finger proteins [96], and is associated with human variation in height [97].

The chromosome 12 duplicon at 7.2 Mb is located 3.3 kb upstream of PEX5 in a potential promoter region. PEX5 is a gene responsible for recognizing PTS proteins in the peroxisome [98]. Defects in PEX5 cause one of several peroxisome biogenesis disorders, accompanied by reduced plasmalogen biosynthesis in the brain [99,100]. Plasmalogen is a lipid that is abundant in myelin, and peroxisome dysfunction leads to demyelination and axon degeneration in the central nervous system [101]. Somatic mutations in a PEX5 promoter could lead to situations in which some neurons are myelin-deficient, causing aberrant signaling. Demyelination has been previously suggested as a pathogenic mechanism in BD [102], and an association between BD and multiple sclerosis (a demyelinating disease) have been observed [103,104]. Valproate treatment for BD appears to change the behavior of the peroxisome in neurons [105].

#### rs4988327 in RA (6)

The scatter plot for this SNP is shown in Figure 3.

LRP5 is a member of the canonical WNT5a signaling pathway that is initiated by IL6 in rheumatoid synovial fibroblasts [106]. LRP5 is also associated with bone mineral density and with susceptibility to osteoarthritis [107,108].

#### rs11010908 in T2D (0)

While there are no characterized genes in the duplicons, two of the duplicons are adjacent, spanning a 370 kb region that includes the genes ANKRD26, YME1L1, MASTL, and ACBD5. ANKRD26-knockout mice develop hyperphagia-induced obesity and insulin resistance [109], as might be expected for a gene associated with T2D.

#### rs295470 in CAD (5)

The function of ACTG1 appears to be the maintenance of the actin cytoskeleton [110]. A muscle-specific ACTG1-knockout leads to progressive myopathy [111]. Conversely, injection of a human ACTG1 construct (but not constructs based on ACTC1 or ACTG2) into adult rat cardiomyocytes caused a cessation of beating, suggesting a dominant negative effect of overexpression of ACTG1 [112]. ACTG1 appears to play an important role in the structure and normal function of striated muscle [111,113].

RBP2 cDNA is down-regulated by low density lipoprotein, which may be relevant to CAD [114]. RBP2 participates in the uptake and/or metabolism of vitamin A, which is converted to retinol. Low plasma retinol is associated with coronary events [115].

#### rs2122231 in BD (0) and HT (0)

rs2122231 is located within a region of human ERV9 retroviral sequence. Gene conversion between this sequence and other ERV9 sequence could change ERV9 expression behavior. Variation in ERV9 expression has been associated with psychiatric disorders, including BD and schizophrenia [116,117].

ERV9 long terminal repeat (LTR) sequence also appears in the promoter of the beta globin gene [118]. Disruptions of ERV9 expression could affect beta globin transcription, providing a plausible link to HT. There are many ERV9 LTR sequences in the human genome; in the absence of evidence that this particular region is responsible for ERV9 expression, the evidence level for these associations is 0.

#### SNP_A-1948953 in HT (3) and BD (3)

LNX proteins including LNX1 interact with members of the Notch signaling pathway that could affect the formation of neuronal cell shape and synaptic connections in the brain [119]. LNX1 interacts with CAST in neurons, and CAST is associated with neurotransmitter release [120]. These properties of LNX1 may be relevant for BD.

LNX1 binds with CXADR, the coxsackievirus and adenovirus receptor [121]. Coxsackievirus seroprevalence has been associated with HT [122].

Interestingly, LNX1 RNA is a much closer match to the SNP_A-1948953 duplicon than the LNX1 DNA; there are gaps in homology that coincide with the LNX1 introns.

#### rs9839841 in CD (4)

**Figure 6:**
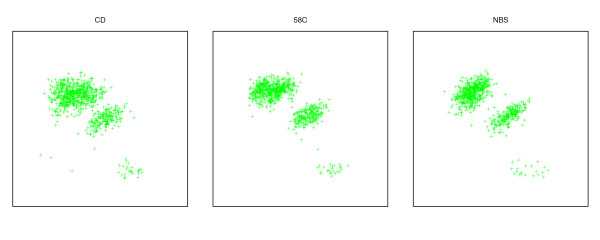
**Cluster plot for males at the rs9839841 locus**. The populations are CD (788 males), 58C (752 males), and NBS (720 males). Note the higher spread of the data points in CD.

The duplicon for this SNP is on the Y-chromosome, suggesting that gene conversion should be observed only in males. The rs9839841 SNP is a C/T polymorphism on chromosome 3. The corresponding Y-chromosome locus has a 35 bp flanking sequence that is identical to the chromosome 3 sequence containing the T allele. As a result, the microarray will show a base intensity for the T allele that is higher in males than in females. One should thus interpret the scatter plots and clustering results with caution, as they may be influenced by the relative frequency of each gender in the population. In support of a true CD association at this locus for males, Figure 6 shows a scatter plot limited to males for the CD, 58C and NBS populations. The CD population shows a higher spread despite having approximately the same number of data points as each control population.

RFTN1 modulates T-cell signals, particularly Th_17_, and influences the severity of autoimmune responses [123]. RFTN1 is also needed for B-cell receptor signal transduction [124]. CD and some other chronic inflammatory diseases are mediated by Th_17 _cells [125,126].

#### rs4850057 in T2D (6) and BD (4)

UNC13B expression is reduced in pancreatic beta cells of rat models of T2D [127]. Conversely, overexpression of UNC13B amplifies insulin exocytosis [127]. These results are directly relevant to T2D in which insulin exocytosis is dysregulated [128,129].

UNC13B also modulates neurotransmitter release in neurons [130-132], a pathway relevant for BD.

#### The HLA region in T1D

It is difficult to separate a conversion signal from the broader association signal for T1D in the MHC region; the MHC region on chromosome 6 has extensive association with T1D [11]. Recent high resolution studies have identified an association signal at the HLA-B locus (but not the HLA-C locus) that is independent of the MHC class-II loci [79]. HLA-C has been linked with T1D when considered in combination with KIR genes that are expressed in natural killer cells [133,134].

There are many plausible ways that disruption of an immunity-related gene could modulate T1D pathogenesis. Gene conversion provides additional candidate hypotheses. For example, gene conversion in the duplicons associated with rs389600 could lead to disruption of HLA-G expression. HLA-G expression is immunoprotective in pancreatic islets [135]. An association between the HLA-G region and T1D has previously been observed [136].

### Significance of stringent test associations

The ways that the identified genes appear to be relevant to the corresponding disease are diverse. This diversity makes it difficult to formally quantify the significance of the noted associations. In particular, it might be that any sample of genes from duplicated regions leads to many associations with disease pathways if the literature is examined to sufficient depth. To eliminate this possibility, and to quantify the degree of 'background' association one would expect, I conducted a mock association study.

In the mock study, I identified ten SNPs for each disease. The SNPs were chosen to reside on known segmental duplications from the segmental duplication database. A chi-squared statistic comparing the distributions of AA/AB/BB genotypes in controls and in the disease samples was computed, and the ten SNPs that minimized this statistic were chosen. (The selected SNPs for a disease sample are therefore those whose genotype distributions are closest to the controls.) For each disease I searched for disease associations using the literature in the same way that associations were sought for SNPs selected by the various filters. The details are presented in Tables S15 and S16 in Additional file 1.

The hypothesis being tested is that the associations of the stringent test and the mock test differ in the degree of association to the corresponding disease. The rate at which known evidence was found in the stringent test and the mock study is summarized in Table 6. SNPs in the MHC region for T1D were excluded. A Fisher's exact test of the difference between the stringent filter and mock study at evidence level three gives *P *< 10^-9^. Even if one limits the stringent test results to SNPs belonging to duplicons in the segmental duplication database, a Fisher's exact test at evidence level three gives *P *< 10^-8^. There are consistent disease associations for 22 of the 28 identified instances, and one can reject the null hypothesis that the observed associations are random.

**Table 6:** Comparison of the stringent and mock tests.

		Strength of evidence						
Test		6	≥5	≥4	≥3	≥2	≥1	0
Mock	(/70)	6 (4)	6 (4)	7 (5)	11 (8)	13 (9)	16 (11)	84 (59)
Stringent	(/28)	21 (6)	36 (10)	54 (15)	79 (22)	79 (22)	79 (22)	21 (6)
	(/16)	31 (5)	50 (8)	62.5 (10)	87.5 (14)	87.5 (14)	87.5 (14)	12.5 (2)

Percentage (count) of SNPs with evidence in the various categories for the mock and stringent tests. The second row for the stringent test limits the analysis to SNPs belonging to a duplicon in the segmental duplication database.

### A permutation test

Another way to assess the significance of the stringent test associations is via a permutation test. By switching the labels of cases and controls with probability 0.5 and applying the stringent test conditions, one can test the null hypothesis that the distribution among cases relative to controls is the same as the distribution of controls relative to cases.

In order to perform this test without manually checking for homology, I limit the analysis to associations in regions of at least 90% homology identified by the segmental duplication database. SNPs in the MHC region for T1D are excluded. With those limitations, there are 16 SNP/disease pairs satisfying the original stringent test. Switching the labels of cases and controls for each disease and SNP yields five qualifying SNP/disease pairs.

Based on this information, it is possible to approximate the permutation test distribution as a binomial distribution with *N *= 21 and a probability of 0.5. The probability *p *that one would observe at least 16 associations under such a distribution is 0.013, allowing us to reject the null hypothesis.

### The relaxed filter

Seventeen stringent-filter SNPs with homology sufficient to satisfy the segmental duplication database constraints are also returned by the relaxed filter. 65 additional instances covering 50 distinct SNPs survive the relaxed filter. Four of these SNPs are among those identified (for other diseases) using the stringent filters. Four additional SNPs are distinct from those identified by the stringent test, but reside in the same duplicons as SNPs from the stringent test. This data is summarized in Table 7. *P *values for these associations are given in Tables S2 and S3 in Additional file 1.

**Table 7:** Additional SNPs identified using the relaxed filter.

SNP	Disease(s)	Characterized genes in duplicons
rs10147986	CD	(40 duplicons)
rs10502407	BD	[CIDEA]
rs10896468	CAD	OR8U8, OR5M8
rs11010995	RA	-
rs11028186	RA	ALG1L, ASNS, [ZNF195], [FAM86B2], [DEFB10P1], [DEFA5], [ZFYVE20]
rs11053044	T2D	ALG10, ALG10B
rs11118278	CAD	CR1L, MCP
rs1192923	HT	[ORAOV1]
rs12227938	BD, CAD, T1D	ALG10, ALG10B
rs12256867	T2D	ZNF33A, ZNF37A, ZNF33B, ZNF37B, [ZNF25]
rs12413153	CAD	DDX18, BTBD15, WDR22, [IBRDC2]
rs1291361	BD	HTR7, HTR7P, [HEBP1]
rs1404223	CAD	-
rs17080801	T2D	PARP4, TPTE2
rs17230081	T2D	ORM1, ORM2
rs17636964	CD	IPMK
rs17645907	T2D	[POMZP3]
rs1842055	CAD	-
rs1868584	CAD, HT, RA, T1D	ROCK2, CGGBP1, [ZNF654]
rs2120273	BD	-
rs2236014	BD	MTRF1L, [FBX05]
rs2515832	RA	MAGEA12, CSAG1, MAGEA2, MAGEA3, TRAG3,
		[MAGEA6]
rs2523544	T1D	DHFRP2, DHFRL1, DHFR, PSMA8, [HLA-B], [MSH3], [NSUN3]
rs2617729	CD, T2D	ZNF761, ZNF765, ZNF813, [ZNF331]
rs330201	CAD	MRPL10, [LRRC46], [OSBPL7]
rs3858741	BD, CD, HT	PSPC1, [TUBA3C]
rs4318932	CD	TYW1, TYW1B, [STAG3L4]
rs4453734	CAD, RA	-
rs4473816	RA	[GSPT2]
rs4532803	BD, HT	ELA3A, ELA3B, [HSPC157]
rs4545817	BD	ALG1, FAM86A, [COL6A4P2]
rs4881702	BD	-
rs500192	BD, T1D	TBL1XR1
rs5946541	BD	[BAGE]
rs6427130	RA	XCL1, XCL2
rs6463213	BD, T2D	RBAK, RNF216L, XKR8
rs6744284	BD	UGT1A3 -UGT1A10
rs6945984	RA	CYP3A4, CYP3A7, [CYP3A5]
rs7259082	CAD	ZNF737, M74509, ZNF66
rs7549545	BD	[IER5]
rs7677996	T1D	[UGT2B7]
rs7808342	BD	-
rs940331	T2D	[ZNF735], [ZNF716]
rs9551988	T2D	PSPC1, [TUBA3C]
rs9624808	T1D	LRP5, LRP5L
rs9665670	BD, CAD	[PDSS1]
rs9775226	CD, HT	(40 duplicons)
SNP_A-1797773	BD, CD	VPS35, [ORC6L]
SNP_A-1817967	CD	FAM22A
SNP_A-1858955	RA	GUSBL1, GUSBL2, SMA4, GUSBP1, [RGL4]

Genes in square brackets are outside the duplicons, but a duplicon is at most 30 kb upstream of the gene. Genes for two SNPs having 40 duplicons each are omitted.

By design, the gene associations identified solely by the relaxed filter may include false positives. Nevertheless, several of these associations appear to be plausible for the disease(s), and are promising candidates for further study.

The region containing rs10502407 in chromosome 18 has known associations with bipolar disorder. GNAL, and possibly other genes in this region, are subject to epigenetic regulation, and constitute potential susceptibility genes for BD and schizophrenia [137].

rs3858741 is identified as a gene conversion locus for BD, CD and HT and rs9551988 is associated with T2D. These two SNPs are within the same duplicon. The discussion of rs9551988 for the stringent filter analysis covers the BD, HT, and T2D associations. The NO pathway also appears to be important for CD [138,139].

ALG10B is associated with HT in the stringent filter. The association with CAD in the relaxed filter can also be attributed to elongated QT intervals, as can the association with T1D [140]. ALG10B also appears to modulate K^+ ^current in neurons [141], making the link to BD plausible. rs11053044 is identified as a gene conversion locus in T2D; rs11053044 falls within the ALG10 duplicon. Elongated QT intervals are also observed in T2D [142]. Variants of the pore-forming alpha-subunit potassium channel gene KCNQ1 are associated with reduced insulin secretion and T2D [143], and with forms of the long QT-syndrome [143]. VPS35 is associated with BD and CD. VPS35 appears to regulate Wnt signaling [144]. Wnt signaling is important for the proper structure of the absorptive epithelium of the small intestine [145], a plausible link with CD. The Wnt pathway is also associated with BD [146].

The SNP rs9624808 is identified in T1D by the relaxed test; rs9624808 is in same duplicon as rs4988327. LRP5 has been identified as a susceptibility locus for T1D [147,148].

The SNP rs1291361 associates HTR7 and HEBP1 with BD. HTR7 is a serotonin receptor that mediates impulsive behavior [149], and appears to have variants associated with schizophrenia [150]. HEBP1 appears to function in the brain's response to oxidative stress [151].

PARP4, associated with T2D, is a DNA repair molecule involved in nick sensing [152].

ROCK2, associated with CAD, HT, RA, and T1D, is involved in various functions including actin cytoskeleton organization, and abnormal activation of the ROCK pathway has been associated with CAD and HT [153].

DHFR, associated with T1D, converts dihydrofolate into tetrahydrofolate, a necessary step for the de-novo synthesis of purines. See Figure 4 and the discussion of rs9378249, which is also associated with T1D by the stringent filter.

XCL1 and XCL2 are associated with RA. XCL1 is produced by T cells in RA [154]. XCL1 and XCL2 regulate the movement of cells expressing XCR1 [155], which is upregulated in synovial fluid in RA [156]. The UGT1A molecules, associated with BD, are responsible for metabolizing and/or eliminating a variety of chemicals, including mutagens and toxins [157].

CYP3A4, associated with RA, is involved in vitamin D metabolism [158].

PDSS1 is associated with CAD and BD by the relaxed filter. A germ-line mutation in PDSS1 was identified in two siblings with cardiac disease and mental retardation associated with coenzyme Q_10 _deficiency [159].

### The no-call-only filter

Seventeen stringent-filter associations meet the no-call-only filter condition on the *p *value; see the *p_n _*column of Table S1 in Additional file 1. (Ten of these also satisfy the homology requirements of the no-call-only filter.) Eight relaxed-filter associations meet the no-call-only filter condition; see the *p_n _*column of Tables S2 and S3 in Additional file 1. Table 8 shows the remaining 50 associations covering 37 distinct SNPs. One of these SNPs (rs4471699) is among those identified (for other diseases) using the stringent filter. Nine of these SNPs are among those identified (for other diseases) using the relaxed filter. This data is summarized in Table 8.

**Table 8:** Additional SNPs identified using the no-call-only filter.

SNP	Disease(s)	Characterized genes in duplicons
rs10238378	BD	-
rs10485575	BD	SNX5, ANO4
rs10768666	RA	HCCA2, KRTAP5-8, KRTAP5-3, [KRTAP5-2], [KRTAP5-1], [KRTAP5-5], [KRTAP5-9], [KRTAP5-10],
rs10811497	BD	IFNA4, IFNA7, IFNA10, IFNA14, IFNA16, IFNA17, IFNA21, [IFNW1]
rs10896468	BD, CD, T2D	OR8U8, OR5M8, [OR5M3], [OR5M9]
rs11228904	BD, HT, T1D	TRIM48, TRIM53
rs11583656	HT	MYPT2, [UBE2T]
rs1191684	BD	[PAX8]
rs12428824	BD	ENPP3, CTAGE4, CTAGE6, [OR2A7], [OR2A20P], [OR2A4]
rs1421867	T1D	-
rs1708080l	BD, HT	PARP4, TPTE2
rs17310770	T2D	ROPN1, ROPN1B, CCDC14
rs17423694	HT	[NBPF11]
rs17636964	BD, RA, T2D	IPMK
rs1809667	T1D	HCCA2, KRTAP5-2, KRTAP5-8, KRTAP5-3, KRTAP5-10, KRTAP5-11, KRTAP5-7, [KRTAP5-1], [DUSP8], [KRTAP5-5], [KRTAP5-9]
rs1819829	HT	CES7, [CES1]
rs1820450	RA	GPC5, [GOLGA8B]
rs1868584	BD	ROCK2, CGGBP1, [ZNF654]
rs193017l	T1D	PCDH15
rs2039945	T2D	-
rs2804672	HT	HSD17B7, HSD17B7P2, CDC10L
rs3864439	BD	DPY19L2, DPY19L2P1, DPY19L2P4, [DPY19L1], [STEAP1]
rs4236384	RA	SLC29A4, TNRC18
rs4318932	T2D	TYW1, TYW1B, [STAG3L4]
rs4471699	BD, T2D	SULT1A3, GIYD2, BOLA2, IMAA, CORO1A, MLAS, SMG1, [UQCRC2], [NPIPL3]
rs4532803	CAD	ELA3A, ELA3B, [HSPC157]
rs4545817	HT	ALG1, FAM86A, [COL6A4P2]
rs584630	BD	ZNF33A, ZNF33B, ZNF37A, ZNF37B, [ZNF25]
rs649483l	RA	FMN1
rs6510085	RA	ZNF419, ZNF773, [ZNF772], [ZNF549]
rs651263l	CAD	-
rs731999l	BD, CAD, HT, T1D, T2D	CENPI
rs8182488	T1D	ZNF765, ZNF761, [ZNF813]
rs9948005	BD	FAM38B
rs9976299	RA	ITGB2
SNP_A-1817967	CAD, CD	-
SNP_A-1858955	CD, BD	GUSBL1, GUSBL2, SMA4, GUSBP1, [RGL4]

Genes in square brackets are outside the duplicons, but a duplicon is at most 30 kb upstream of the gene.

Beyond the SNPs already identified by the relaxed filter, the following no-call-only filter associations appear to be promising candidates for future study.

SULT1A3 in BD and T2D. Impaired sulfation has been linked with various neurological diseases [28,160]. Sulfoconjugation of monoamines via SULT1A3 occurs within the brain, and could represent an important detoxification pathway [28,161]. SULT1A3 is important for the degradation of dopamine in neurons [162], and dopamine dysregulation has been linked with both BD [163] and T2D [164].

TRIM48 and TRIM53 in BD, CD, and T2D. TRIM proteins such as TRIM48 are thought to function during the cellular response to viral infection [165].

CENPI in BD, CAD, HT, T1D, and T2D. CENPI is located on the X chromosome, and is essential for proper segregation during mitosis [166]. Disruption of CENPI results in daughter cells having extra/missing chromosomes [166].

HCCA2 (also known as MOB2) in RA and T1D. HCCA2 appears to be important for proper segregation during mitosis [167,168].

MYPT2 in HT. MYPT2 is expressed in the heart and skeletal muscle where it dephosphorylates myosin and is involved in muscle contraction [169,170]. Note that the matching duplicon is on the Y chromosome, meaning that somatic gene conversion could only happen in males.

GPC5 in RA. GPC5 expression appears to be reduced in arthritis [171] and GPC5 is located within a quantitative trait locus for arthritis [172]. A SNP within GPC5 appears to be significant for parovirus-induced arthritis [173]. Polymorphisms in GPC5 also appear to be associated with the response of multiple-sclerosis patients to interferon beta therapy [174], and GPC5 appears to be a risk factor in multiple sclerosis [175].

HSD17B7 in HT. HSD17B7 catalyzes the conversion of estrone to estradiol [176], and also is involved in cholesterol biosynthesis [177]. Estradiol treatment lowers blood pressure in hypertension [178-181]. Disruption of HSD17B7 could lower endogenous estradiol concentrations leading to an increase in blood pressure.

DPY19L2 in BD. In C. elegans, the DPY19 gene is required to properly polarize and orient migrating neuroblasts during development [182].

ITGB2 in RA. The ITGB2 gene encodes the CD18 adhesion molecule present on several kinds of immune cells. CD18 expression is upregulated in macrophages and T-cells in the peripheral blood and synovial fluid of RA patients [183,184].

### Cluster plot artifacts

The 58C DNA samples were obtained from cell lines, while the other samples (including the NBS control sample) were obtained directly from blood cells [11]. Genomewide, the samples were statistically similar [11]. Nevertheless, it is conceivable that certain SNPs are systematically affected by the procedures used to establish cell lines. A systematic bias that reduces the no-call rate at a SNP in the 58C population could make other populations appear to have high no-call rates at the SNP relative to the combined controls. A significant difference between the 58C and NBS populations in cluster positions for a SNP could be an indicator of such a bias. At the same time, one cannot exclude the possibility that the reasons for this bias may themselves be related to gene conversion. For example, a cell that has undergone a conversion-induced mutation at a locus may not be viable as a cell-line founder, meaning that only cells with unmutated sequence at that locus will be present in the cell-line samples.

A small number of individuals in the WTCCC data generated outlying low-intensity points at multiple loci in the CAD/RA/NBS cohorts, a probable artifact of different procedures for those cohorts [11]. High no-call rates can also occur at a locus with copy number variation, where there are typically more than three clusters. I therefore visually examined all cluster plots for SNPs identified by the various filters, looking for clear examples of any of these three patterns.

The results are summarized in Table 9. For the stringent filter rows labeled with a 58C disparity, the no-call rate for 58C is less than one third of that for NBS. Four of the seven stringent filter SNPs (rs12070036, rs12381130, SNP_A-1797773, rs9257223) have significantly higher no-call rates than the NBS population alone (*P *< 0.005 for a one-sided chi-squared test). The remaining three SNPs have no-call rates that are not significantly difierent from the NBS population (*P *> 0.05). The *P *value for the stringent filter comparison with the mock study remains below 10^-8 ^at evidence level three even if all stringent filter SNPs in Table 9 are excluded.

**Table 9:** SNPs with anomalous cluster plots.

Filter	SNP	Disease(s)	Cluster plot feature
Stringent, relaxed	rs10502407	CAD, T2D, BD	58C disparity
Stringent	rs11010908	T2D	58C disparity
Stringent	rs12070036	BD	58C disparity
Stringent	rs12381130	T1D	58C disparity
Stringent	rs295470	CAD	58C disparity
Stringent, relaxed	SNP_A-1797773	T2D, BD, CD	58C disparity
Stringent (MHC in T1D)	rs9257223	T1D	58C disparity
Relaxed	rs11028186	RA	58C disparity
Relaxed	rs12256867	T2D	58C disparity
Relaxed	rs1404223	CAD	58C disparity
Relaxed	rs17230081	T2D	58C disparity
Relaxed	rs1842055	CAD	58C disparity
Relaxed	rs330201	CAD	58C disparity
Relaxed, no-call	rs4318932	T2D	NBS/CAD/RA disparity
Relaxed	rs4473816	RA	58C disparity
Relaxed	rs7259082	CAD	58C disparity
Relaxed	rs9665670	BD, CAD	58C disparity
Relaxed, no-call	SNP_A-1817967	CD	58C disparity
No-call	rs10238378	BD	58C disparity
No-call	rs10485575	BD	58C disparity
No-call	rs10811497	BD	58C disparity
No-call	rs12428824	BD	58C disparity
No-call	rs1421867	T1D	more than 3 clusters
No-call	rs1819829	HT	NBS/CAD/RA disparity
No-call	rs2039945	T2D	58C disparity
No-call	rs2804672	HT	NBS/CAD/RA disparity
No-call	rs6512631	CAD	58C disparity

The SNP rs7761068 was considered in Figure 5 for the analysis of rs9378249. The proportion of low-intensity individuals at rs7761068 does not segregate with the RA, CAD and NBS populations, and the 58C and NBS populations have similar intensity distributions, suggesting that the observed effect at rs7761068 is not artifactual.

Since each cohort has a different proportion of males, a duplicon on a sex chromosome could skew the cluster plot results in a population specific way. Such skew is clear for rs9839841, where a duplicon is on the Y chromosome, and where 94% of the no-calls in CD are for males. Measurements of the male proportion of no-calls for all of the other stringent filter SNPs were close to the proportions in the population as a whole (data not shown). This observation excludes the possibility that a probe sequence for these SNPs is absent from the reference human genome yet occurs frequently in the population on a sex chromosome.

### Linkage

In the present study, concordant observations at several adjacent SNPs were not expected [10], and the analysis did not require such observations. Looking at the 28 SNPs identified by the stringent filter in Tables 2 and 3 retrospectively, one can look for evidence of linkage in the form of a significantly increased no-call rate at SNPs adjacent to the target SNP. Evidence of linkage at the 28 loci, within the SNP resolution available on the microarray platform, is summarized in Table 10.

**Table 10:** Linkage between stringent-filter SNPs and adjacent SNPs.

Stringent test SNP	Disease	Adjacent SNP	*P*	Comments
rs9551988	HT	rs3858741	3.2 × 10^-12^	1.1 kb away, within same duplicon
	BD	rs3858741	8.9 × 10^-8^	(No linkage for CAD.)
				
rs9378249	BD	rs2596477	3.7 × 10^-7^	22 bp away, within same duplicon
	HT	rs2596477	6.0 × 10^-4^	
				
rs841245	HT	rs12229182	2.5 × 10^-6^	12 kb away, within same duplicon
		rs841636	1.7 × 10^-5^	12 kb away, within same duplicon
				(No linkage at intervening SNP rs10842853)
				
SNP_A-1948953	BD	rs9893203	5.4 × 10^-4^	8.6 kb away
	HT	rs9893203	6.8 × 10^-4^	
				
rs11010908	T2D	rs17561365	1.2 × 10^-3^	3.2 kb away, within same duplicon

The *P *value corresponds to a one-sided chi-squared test for an increased no-call rate. *P *values for excluded SNPs were all above 2 × 10^-3^.

These linkage results demonstrate that strong linkage is unusual, and that when it occurs, linkage is typically limited to one neighboring SNP. These results also suggest that linkage is more common in BD and HT than in other conditions.

### Somatic deletion

While the filters discussed previously are designed to identify gene conversion, it is possible that they also capture cases of somatic deletion. Somatic deletion at a SNP locus would be indistinguishable from somatic conversion within the flanking sequence of the SNP. Looking at the stringent filter results, approximately half of the loci have pairs of duplicons within a few megabases of each other on the same chromosome. This pattern could lead to deletions through gene conversion, improper recombination, or due to removal of sequence fragments forming hairpin-like structures [185]. Somatic duplication is also possible. For rs12381130 and rs11010908, there is no disease-related gene within any of the duplicons, while disease-related genes do occur between duplicons. (The LRP5 gene resides on the chromosome 11 interval for rs12381130, and the ANKRD26 gene resides on the chromosome 10 interval for rs11010908.) For rs9378249, the data suggest that there is a somatic deletion of the DHFRP2 pseudogene.

There is another kind of deletion that could give rise to results that might appear like gene conversion. Consider a SNP locus in which there exists a duplicon having 100% sequence identity in the flanking sequence. This duplicon would add to the signal of one of the alleles at the SNP locus.(Cross-hybridization with less that 100% identity is possible, but is ignored here.) Assuming the duplicon is not polymorphic, this additive signal would be consistent across individuals. The positions of the clusters would be different from a situation without such a duplicon, but AA/AB/BB clusters would still be able to be differentiated from one another.

Imagine a disease associated phenomenon in which there is increased deletion of the duplicon (but not the SNP region) due to improper recombination. In such a case, there would be a bias towards a loss of signal for the allele that is present in the non-polymorphic duplicon. This is the opposite bias to what one expects from gene conversion of the SNP region by its duplicon (although as discussed in Additional file 1, for conversion of major to minor alleles, such a bias is still possible).

To investigate this possibility, I re-examined the results of the stringent filter to identify cases where there is (a) 100% identity of the duplicon within the SNP's flanking sequence, and (b) a change in the allele distribution away from the allele in the duplicon. There is one such SNP, namely rs9551988, that accounts for three of the five observations (Table S1) where the allele frequency changes away from the allele in the non-polymorphic duplicon. Given the additional information that the duplicons for rs9551988 are 500 kb away from each other on the same chromosome and in the same orientation, it is reasonable to infer that deletion is the likely explanation for the results observed at this locus.

Now imagine a disease associated phenomenon in which there is increased deletion of the SNP region (but not the non-polymorphic duplicon) due to improper recombination. In such a case, there would be a bias towards a relative loss of signal for the allele that is not present in the non-polymorphic duplicon. This is the same bias that one expects from gene conversion of the SNP region by its duplicon. I therefore re-examined the results of the stringent filter to identify cases where there is (a) 100% identity of the duplicon within the SNP's flanking sequence, and (b) a change in the allele distribution towards the allele in the duplicon. There are four such cases, namely rs669980, rs935019, SNP_A-1797773, and rs9839841. Of these, only rs935019 represents a case with nearby aligned duplicons on the same chromosome. For rs935019, variation in copy number has been observed in cloning experiments [65], suggesting that deletion is the most likely explanation for this locus.

An additional example was observed during the examination of SNPs using BLAST to determine whether they reside in a region with homology elsewhere in the genome. rs2812 met the stringent filter conditions for CAD except that it did not reside in a duplicated region. Nevertheless, a 400 bp duplicon occurred both upstream and downstream of rs2812, together spanning a 2 kb region including the SNP. rs2812 is located within the PECAM1 gene, which has previously been associated with CAD [186-188]. Out of approximately 250 SNPs that were examined in this way, rs2812 was the only one for which this kind of duplication pattern was observed. Nevertheless, the present study was not designed to identify such patterns, and additional longer-range (or inter-chromosomal) duplication that increases the likelihood of sequence deletion may exist.

### Known de-novo non-allelic conversion sites

Five pairs of genes have been identified as loci of de-novo germ-line gene conversion between non-allelic regions, leading to a disease phenotype [1]; see Table 11. If these conversion events are frequent enough to be noticed even in the germline, then such loci may be likely to be sites of relatively frequent somatic conversion. I therefore examine SNPs located in duplicons related to these gene pairs to determine whether the cluster plots support this hypothesis.

**Table 11:** De-Novo conversion events in disease [1].

Disease	Donor	Acceptor
Atypical haemolytic uraemic syndrome	CFHR1	CFH
Congenital adrenal hyperplasia	CYP21A1P	CYP21A2
Neural tube defects	FOLR1P	FOLR1
Hereditary persistence of fetal haemoglobin	HBG2	HBG1
Shwachman-Diamond syndrome	SBDSP	SBDS

I consider all SNPs appearing in one of the two duplicons shared by the two genes. Coverage is limited by the resolution of the microarray. In fact, no SNPs are available for the CYP21A1P/CYP21A2 genes. For the SBDSP/SBDS pair, there are four almost-contiguous segmental duplications in the segmental duplication database, spanning just over 500 kb. I consider all SNPs in all of the four duplicons. I visually inspected the cluster plots for the SNPs in the corresponding duplicons. The target pattern is one in which for every population (including controls) there is a substantial number of points between clusters. The results of the visual cluster plot analysis are summarized in Table 12. The visual analysis is supported by the WTCCC quality control procedures: for seven of the nine identified SNPs (all except rs6578592 and rs1465306) the SNP was excluded for quality control reasons such as departure from HWE in the control population. (One additional SNP, rs1880278, was also excluded for quality control reasons but did not show features predicted for gene conversion.)

**Table 12:** Possible conversion in duplicons for genes previously observed to have undergone germ-line conversion.

Genes	Number of SNPs in duplicons	SNPs showing possible conversion
CFHR1/CFH	7	rs395998, rs413979
FOLR1P/FOLR1	5	rs1540087
HBG2/HBG1	3	rs6578592
SBDSP/SBDS	38	rs4717344, SNP_A-1849003, rs4718487, rs1465306, rs2003206

Events are identified by visually inspecting cluster plots for all SNPs in the region.

Given the small sample size and sparse coverage of the duplicons, the results of Table 12 are suggestive, but far from definitive.

### Disease-specific patterns

Based on the SCE data, RA was predicted to be a local disease. Four SNPs that are associated with RA (rs4988327, rs10768666, rs4236384, rs9976299) have cluster plots in which RA alone has an increased number of no-calls. When other disease populations have correlated behavior, the RA population sometimes appears to remain close to the control population, as exemplified in Figure 5. In contrast, no other disease population has an associated SNP for which that population alone has an increased no-call rate.

These results are broadly consistent with a view of RA as a local disease, and of the remaining diseases as global diseases. The distinction is not clear-cut, however, since there are RA-associated SNPs with no-call behavior that is similar across multiple diseases.

An alternative interpretation of the distinctness of RA is based on the observation that lymphocytes may be the initiators of RA pathogenesis. Since lymphocytes are the cells being genotyped, lymphocyte-specific autoimmune dynamics could amplify the signal attributable to pathogenic mutations. For example, a mutation in a T cell that leads to cell activation and replication would substantially increase the population of cells exhibiting the mutation. Of the four SNPs showing RA-specific spread in the cluster plots, rs9976299 is notable for being within the ITGB2 gene which encodes the CD18 adhesion molecule. CD18 expression is upregulated in macrophages and T-cells in the peripheral blood and synovial fluid of RA patients [183,184].

BD and HT co-occur at four different stringent-filter SNPs. Three of these SNPs display similar linkage patterns with neighboring SNPs for both BD and HT. These factors suggest that BD and HT may have a common ultimate cause that is different from the other five diseases. A general similarity between HT and BD has previously been identified using a classification algorithm over the same WTCCC data set [189]. Individuals with BD have a more than twofold increased risk of HT [190].

## Discussion

Based on prior data for loci such as IDS [3,4], disease related genes were sought in one of several duplicons, only one of which contains the identified SNP. For 8 out of the 28 stringent filter SNPs, the disease related gene is on a duplicon not containing the SNP, emphasizing the importance of examining all duplicons. Such genes would not be identified using a conventional association study.

Confounding factors could perturb cluster plots, potentially leading to false associations. Loci that did not meet the WTCCC quality control requirements have been excluded. The WTCCC reports a disparity between the NBS/RA/CAD cohorts and the other cohorts for some SNPs [11]; such disparities are rare among the SNPs meeting the filter conditions (Table 9). Additional quality control issues not identified by the WTCCC are possible. Nevertheless, it is hard to imagine how a quality control artifact could lead to population-specific effects that correlate with disease related genes.

Copy-number variation can be discounted as a general explanation for the observed phenomena, since none of the stringent test SNPs (and only one of the no-call SNPs) showed more than three clusters. Further, few of the stringent filter SNPs are within known CNV loci (Additional file 1). Even if copy number variation was the mechanism responsible for some of the present results, the results would still be interesting as novel cohort-specific associations.

The present paper provides support for the hypothesis that many complex diseases are caused in part by somatic mutation in regions with homology elsewhere in the genome. Diseases such as cancer often display gross karyotypic changes that could be due to improper recombination between nonallelic homologous regions in somatic tissue. Because detection of somatic mutations is technically much more demanding than that of germline mutations, somatic gene-conversion events in cancer have probably been underestimated [1].

Some puzzling epidemiological features of autoimmune diseases are consistent with a somatic mutation hypothesis. Association with viruses can be explained by the mutagenic actions of those viruses. Associations of autoimmune disease with higher latitudes has been hypothesized to relate to lower vitamin D levels [191]; vitamin D is associated with lower rates of double-strand breaks [192] and with protection from viral infections [193]. Complex inheritance patterns spanning multiple diseases would result from a common underlying genetic susceptibility based on sequence homology, combined with stochastic effects such as tissue-specific viral infection.

In order to be identified as a conversion region in this study, the region must contain a locus that is within the SNP repertoire of the microarray chip. A substantial amount of somatic gene conversion might affect loci with alleles that are fixed in the population. If so, alternative platforms will be needed to detect such conversion. It is likely that there is additional disease-specific somatic gene conversion that the present study has not detected even among the covered SNPs. Spread in the cluster plots might not be apparent if a particular disease-causing somatic mutation was rare enough that the perturbation was small relative to experimental variation.

On the other hand, common gene conversion events might preferentially include SNP loci. If a conversion event is common in somatic tissues, it may also be relatively common in the germ-line. If the germ-line event is not deleterious, a polymorphism could result. The consequences of somatic and germ-line changes are different, and a somatic mutation may cause disease where a germ-line change does not. For example, a somatic mutation may result in a novel protein that is immunogenic. Alternatively, some of the loci associated with a conversion event may be phenotypically neutral, and these may lead to polymorphisms as a result of partial conversion events in the germ-line.

The phenotype of a somatic mutation is likely to be very different from the phenotype of a germ-line mutation. Outside of cancer, there is very little data about phenotypes associated with somatic mutations. It is therefore dificult to correlate the observations of this paper with existing knowledge about somatic mutation. Correlations with genomewide studies of disease associated polymorphisms are possible in principle. However, given the methodologies used in those studies (for example, requiring multiple concordant SNPs [11]), it is not expected that correlations will be found given the absence of linkage disequilibrium for gene conversion [10].

It may well be that somatic gene conversion is, in some cases, a normal adaptive phenomenon. Such effects might be detectable using SNP microarrays by examining the intensity plots directly without employing a calling algorithm. The quality control protocols of SNP array studies typically exclude loci where the called allele frequencies depart from HWE in the control population, which would exclude loci for which somatic gene conversion was common. It may be worth re-examining such loci, particularly those in duplicated regions.

The present report suggests that somatic gene conversion is associated with mutations and genomic rearrangements that lead to disease. Working backwards, one could generate hypotheses for further study by identifying genomic regions with high degrees of homology that contain disease-relevant genes. For example, the BRCA1 gene that is involved in DNA stability and repair pathways [194] itself contains a segmental duplication that includes part of the gene and its promoter region [195,196]. Some BRCA1-related cancers appear to be caused by gene conversion events in individuals carrying one mutant BRCA1 allele [197]. Once BRCA1 function is compromised, gene conversion and rearrangement at other loci may become more frequent.

Gene conversion could be a cause of the disease phenotype, or it could alternatively be a side-effect of an underlying disease-causing genetic disorder with no direct bearing on the phenotype. The fact that disease associations are found for most of the stringent filter SNPs is strongly suggestive of a causative link in which the specific conversion events are the proximate causes of the phenotype.

I have used the output of the Chiamo algorithm without modification. Spread is inferred from a high number of no-call results at a locus. While this method of inferring spread appears to have been effective, more effective methods might be possible. Methods could measure suitably defined 'spread statistics' given allele intensity distributions for several populations.

The success of the analysis supports the hypothesis suggested by the sister chromatid exchange studies that DNA in lymphocytes undergoes similar transformations to DNA in tissues affected by disease. In principle, it may be possible to test for various somatic mutations using a blood sample. Specialized microarrays could be developed to detect specific sequences resulting from common somatic mutations. 

Several important questions remain. The present study does not allow the quantification of risk associated with any particular gene conversion locus. Even the identification of which individuals have substantial conversion at a locus is approximate. Locus-specific experimental studies of conversion frequencies in health and disease are needed.

The present study also does not quantify the degree of conversion necessary to cause disease. In lymphocytes, for example, mutations in a very small number of cells could cause disease if those cells undergo clonal expansion. In other tissues, many cells might need to be mutated before tissue function is compromised. Stem cell mutations (which may be relatively common due to frequent mitosis) could lead to a regular inflow of mutated cells.

Disease associations with a number of specific genes have been suggested by the present work. Changes at these loci in somatic tissues may represent the proximate cause of disease. Nevertheless, the ultimate cause of disease is the factor that causes the DNA damage. Environmental factors are likely to play a significant role. The association of the folate-dependent thymine nucleotide synthesis pathway with several diseases, together with an increase in the frequency of SCEs under methotrexate treatment [198], also suggests another kind of ultimate cause in which impaired DNA synthesis leads to homology-driven repair [199].

## Conclusions

That somatic gene conversion may occur frequently has been previously suggested, but progress has been hampered by the technical difficulty of measuring somatic gene conversion on a large scale [1]. The present study is the first to use genome-scale SNP data to infer somatic gene conversion loci in specific populations. For more than 75% of the loci, genes within the locus associate with the corresponding disease in a manner consistent with known gene/disease associations.

Any single association identified in this report should be considered tentative, and subject to experimental confirmation. Nevertheless, taken together, the associations provide compelling evidence that somatic gene conversion and/or somatic deletion at particular loci influence each of the seven studied diseases. The techniques developed are not specific to the WTCCC data, and could be applied to other data sets to identify putative gene conversion for other diseases.

## Abbreviations

BD: bipolar disorder; bp: base pair; CAD: coronary artery disease; DSB: double strand break; HT: hypertension; kb: kilobases; RA: rheumatoid arthritis; SCE: sister chromatid exchange; SNP: single nucleotide polymorphism; T1D: type-1 diabetes; T2D: type-2 diabetes; WTCCC: Wellcome Trust Case Control Consortium.

## Competing interests

The author declares that he has no competing interests

## Pre-publication history

The pre-publication history for this paper can be accessed here:

http://www.biomedcentral.com/1741-7015/9/12/prepub

## Supplementary Material

Additional file 1 **Supporting text and tables**. Detailed information about the identified SNPs, copy number variation, SNP interactions, and the mock study. Click here for file

Additional file 2 **Zip archive containing cluster plots for all SNPs mentioned in the main text**. The 58C and NBS populations are approximatel 1,500 while the other populations are each about 2,000. Click here for file
