# Supplementary material for: Evidence for somatic gene conversion and deletion in bipolar disorder, Crohn's disease, coronary artery disease, hypertension, rheumatoid arthritis, type-1 diabetes, and type-2 diabetes
Source: BMC Med. 2011 Feb 3;9:12. doi: 10.1186/1741-7015-9-12 (PMC3048570; doi:10.1186/1741-7015-9-12)
Supplement: Additional file 1 — Supporting text and tables. Detailed information about the identified SNPs, copy number variation, SNP interactions, and the mock study. [file 1741-7015-9-12-S1.PDF]

Evidence for Somatic Gene Conversion and Deletion in Bipolar Disorder, Crohn's Disease, Coronary Artery Disease, Hypertension, Rheumatoid Arthritis, Type-1 Diabetes, and Type-2 Diabetes

Kenneth Andrew Ross*

Department of Computer Science, Columbia University, New York NY 10027, USA

Email: kar@cs.columbia.edu;

*Corresponding author

Additional File 1

No-Calls in Chiamo

When the converting sequence possesses a B allele, the spread from the AA cluster to the AB cluster will be twice that of the spread from the AB cluster to the BB cluster, as explained in the main text. When A is the more common allele, points between AA and AB will be the most common between-cluster points and will frequently be no-calls. As a result, the B allele frequency will appear to increase among called genotypes. When A is the rarer allele, the higher spread from AA to AB may be counterbalanced by a higher number of AB genotypes relative to AA genotypes. In this case, Chiamo may display a bias in either direction, identifying a smaller number of more distant points as no-calls, or a larger number of less-distant points as no-calls. In some cases, both groups of points may be identified as no-calls, with only a minor resulting bias.

Occasionally, two clusters will appear so close together in a population that Chiamo redefines the clusters for that population. In that case, a subset of points from one cluster will be called as part of the neighboring cluster, leading to very large changes in apparent genotype frequencies. Such effects are apparent when the Chiamo calls are visualized in cluster plots, as illustrated in Figure S1. This kind of mis-clustering creates problematic genotype calls and extremely small p values for chi-squared tests comparing populations. Nevertheless, mis-clustering is a strong indicator of increased dispersion that generates points in the intermediate region between clusters. The identification of this kind of dispersion is

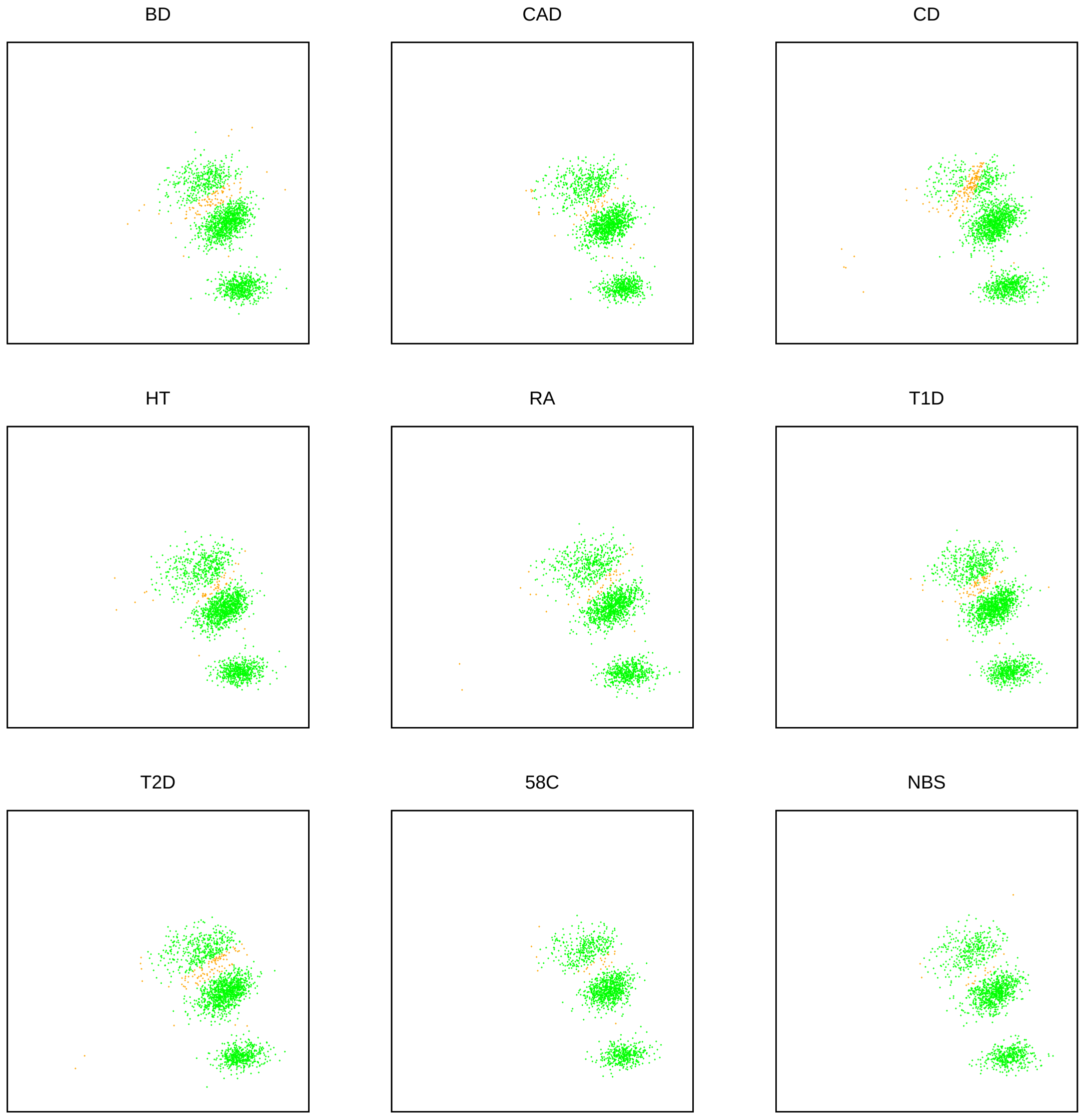

Figure S1: **Cluster plots for SNP rs4471699.** Note the change in cluster boundaries for CD (the no-calls in orange delineate the boundaries).

the present goal. 7 of the 23 stringent filter SNPs show this kind of cluster boundary shift: rs4471699, rs669980, rs11060028, rs3805006, rs9378249, SNP_A-1948953, and rs9839841.

The WTCCC authors suggest that the increase in the frequency of one allele in the called genotypes might lead to a spurious association of that allele with the disease phenotype if there are between-population differences in the cluster shapes [11]. The question of whether the associations are spurious will be answered by the analysis itself. If the effects are spurious, the generated associations will be random and not associated with genes relevant to the disease. In contrast, if the increased no-call rates are due to gene conversion, then the generated associations should target genes that are well-correlated with the disease phenotype.

Details of SNP Associations

p values for the various filter conditions are presented in Tables S1, S2, S3, and S4. In these tables, p_d is the p value for the two-sided chi-squared test for different genotype distributions between cases and controls. p_n is the p value for the one-sided chi-squared test for an increased frequency of no-calls in cases relative to controls. p_h is the p values for the two-sided chi-squared test for HWE in cases. Additional information about allele frequency changes for stringent filter SNPs is presented in Table S1.

Copy Number Variation

Germ-line copy number variation (CNV) might play a role at the SNPs returned by the stringent filter. The TCAG database [26] was consulted to determine whether the SNP is at a known CNV locus. The results are summarized in Table S5. Thirteen stringent filter SNPs are not located in known CNV regions. Of the remainder, most are rare CNVs, and most regions are wide, reflecting the relatively low resolution of current CNV detection methods.

One cannot presently exclude the possibility that there are small-scale CNVs at additional loci that have so far escaped detection. Nevertheless, the complete absence of cluster plots with more than three clusters from the stringent filter SNPs suggests that germ-line copy number variation is unlikely to explain the observed phenomena.

A different kind of copy number variation was considered by Alkan et al. [200]. They used coverage intensity information to identify copy numbers for large (at least 20kb) regions. Their threshold for identity was 95%, meaning that duplicons with more than 95% homology would be considered copies in their analysis. Several of the stringent filter genes were shown to have high copy number in the three individuals

Table S1: **Details of SNPs identified using the stringent filter.**

p_d	p_n	p_h	Disease	SNP	Alleles (major/ minor)	Allele with increased frequency	Allele in opposite duplicon(s)	Change in allele frequency
1.2E-46	1.9E-27	2.0E-45	CD	rs4471699	G/T	G	G	0.100
2.4E-52	1.8E-54	2.2E-61	RA	rs669980	A/C	A	A	0.081
2.6E-02	1.7E-05	7.7E-06	T2D	rs10502407	A/C	A	A	0.009
8.7E-03	1.2E-05	2.5E-07	CAD	rs10502407	A/C	A	A	0.004
8.1E-02	5.3E-09	4.1E-04	CAD	rs12134625	C/A	A	A	0.013
7.2E-02	2.0E-08	4.6E-04	T1D	rs12381130	C/A	C	C	0.004
2.1E-09	2.7E-12	5.0E-11	HT	rs935019	C/T	T	T	0.024
1.0E-12	1.9E-36	3.6E-02	CD	rs11060028	C/T	T	T	0.049
1.3E-04	8.5E-06	1.8E-04	CAD	rs9551988	C/T	T	C	0.021
6.3E-03	1.6E-05	2.7E-04	BD	rs9551988	C/T	T	C	0.002
5.2E-03	1.5E-10	2.1E-04	HT	rs9551988	C/T	T	C	0.004
3.2E-202	2.4E-39	1.2E-159	T1D	rs3805006	C/T	T	T	0.220
5.5E-05	7.8E-07	4.6E-07	CAD	rs295470	A/G	A	A	0.013
1.0E-04	4.9E-06	1.6E-06	HT	rs12227938	A/T	A	A	0.020
7.7E-06	4.1E-15	7.6E-03	T2D	SNP_A-1797773	T/C	T	T	0.042
5.0E-08	7.5E-26	2.7E-01	BD	rs9378249	T/G	T	T	0.028
6.5E-06	5.5E-22	1.7E-01	HT	rs9378249	T/G	T	T	0.022
2.8E-04	1.3E-10	1.6E-01	BD	rs12070036	C/T	T	T	0.040
9.8E-02	1.7E-05	1.4E-04	T2D	rs11010908	C/T	C	C	0.006
1.1E-04	1.0E-11	6.5E-02	RA	rs4988327	A/G	G	A	0.018
2.6E-02	4.9E-10	8.0E-05	HT	rs841245	T/G	G	T	0.018
2.0E-04	9.9E-09	1.8E-09	BD	rs2122231	T/A	T	2T,3A,1-	0.025
2.7E-02	3.3E-13	2.6E-07	HT	rs2122231	T/A	T	2T,3A,1-	0.010
1.0E-14	1.1E-21	2.5E-05	CD	rs9839841	C/T	T	T	0.063
7.5E-16	4.9E-08	9.4E-22	HT	SNP_A-1948953	C/T	C	C	0.044
1.5E-13	3.1E-06	5.3E-20	BD	SNP_A-1948953	C/T	C	C	0.039
8.2E-04	2.2E-07	2.5E-05	T2D	rs4850057	T/C	T	T	0.016
2.5E-05	1.3E-05	1.5E-03	BD	rs4850057	T/C	T	T	0.032

Table S2: p **values of SNPs identified using the relaxed filter (part 1).**

p_d	p_n	p_h	Disease	SNP
3.1E-02	3.8E-04	4.7E-04	CD	rs10147986
3.0E-01	6.3E-05	1.6E-03	BD	rs10502407
2.7E-03	3.1E-03	3.5E-01	CAD	rs10896468
1.2E-03	3.8E-03	3.1E-01	RA	rs11010995
5.0E-02	2.8E-04	2.1E-04	RA	rs11028186
2.4E-01	1.5E-06	8.5E-04	T2D	rs11053044
1.4E-01	2.0E-11	8.3E-03	CAD	rs11118278
2.6E-03	4.7E-14	3.8E-02	HT	rs1192923
1.5E-03	6.8E-04	3.1E-04	BD	rs12227938
8.2E-03	3.2E-03	7.0E-04	CAD	rs12227938
1.6E-04	3.8E-03	5.8E-06	T1D	rs12227938
9.6E-03	2.2E-07	4.6E-01	T2D	rs12256867
2.2E-02	1.0E-03	4.5E-03	CAD	rs12413153
1.4E-01	5.5E-04	6.7E-03	BD	rs1291361
6.3E-02	2.1E-03	6.2E-03	CAD	rs1404223
6.0E-03	1.2E-05	2.0E-01	T2D	rs17080801
5.8E-03	7.7E-06	7.0E-02	T2D	rs17230081
6.6E-03	3.7E-05	5.4E-03	CD	rs17636964
4.4E-04	3.5E-04	3.9E-02	T2D	rs17645907
8.7E-02	1.5E-03	2.6E-03	CAD	rs1842055
4.0E-02	3.3E-03	3.9E-05	CAD	rs1868584
3.1E-01	1.7E-07	2.6E-03	HT	rs1868584
2.0E-01	6.4E-04	9.7E-04	RA	rs1868584
1.4E-01	1.3E-10	1.4E-03	T1D	rs1868584
2.1E-01	1.2E-03	5.1E-04	BD	rs2120273
2.2E-03	5.9E-04	7.2E-07	BD	rs2236014
5.2E-02	1.2E-03	4.9E-03	RA	rs2515832
1.6E-29	1.4E-03	1.0E+00	T1D	rs2523544
2.6E-02	8.7E-05	5.0E-05	CD	rs2617729
1.2E-02	2.7E-05	7.1E-04	T2D	rs2617729
2.7E-03	9.5E-04	3.9E-08	CAD	rs330201
8.8E-03	8.9E-08	5.1E-02	BD	rs3858741
1.0E-02	2.2E-09	4.2E-02	CD	rs3858741
7.4E-03	3.2E-12	4.6E-02	HT	rs3858741

Table S3: p **values of SNPs identified using the relaxed filter (part 2).**

p_d	p_n	p_h	Disease	SNP
3.7E-03	6.5E-06	1.0E-01	CD	rs4318932
3.5E-02	2.1E-03	4.9E-03	CAD	rs4453734
7.0E-02	4.3E-03	7.9E-03	RA	rs4453734
1.9E-02	1.7E-03	1.7E-03	RA	rs4473816
1.3E-02	7.7E-05	6.5E-03	BD	rs4532803
3.0E-02	4.3E-08	1.2E-03	HT	rs4532803
9.4E-03	4.1E-08	8.0E-01	BD	rs4545817
9.1E-03	1.9E-03	9.3E-01	BD	rs4881702
2.5E-02	3.4E-10	1.4E-03	BD	rs500192
2.0E-01	6.0E-07	9.4E-03	T1D	rs500192
6.9E-03	2.9E-03	7.1E-03	BD	rs5946541
7.0E-03	3.8E-05	5.0E-02	RA	rs6427130
7.8E-03	1.7E-07	4.3E-01	BD	rs6463213
6.7E-03	6.5E-05	8.5E-01	T2D	rs6463213
9.6E-03	3.6E-03	6.7E-01	BD	rs6744284
5.8E-03	2.1E-04	6.2E-03	RA	rs6945984
2.5E-01	6.2E-04	9.3E-04	CAD	rs7259082
9.0E-02	1.7E-03	6.1E-04	BD	rs7549545
7.3E-03	4.2E-03	2.1E-02	T1D	rs7677996
8.6E-04	1.1E-11	3.8E-01	BD	rs7808342
9.7E-03	2.8E-03	7.3E-01	T2D	rs940331
4.2E-04	1.6E-04	1.2E-05	T2D	rs9551988
8.1E-03	2.5E-03	9.0E-06	T1D	rs9624808
3.0E-02	2.4E-03	2.9E-05	BD	rs9665670
5.9E-02	9.4E-04	2.8E-04	CAD	rs9665670
1.3E-01	2.8E-03	7.2E-03	CD	rs9775226
1.4E-01	2.0E-03	2.5E-03	HT	rs9775226
2.1E-03	3.1E-17	1.1E-02	BD	SNP_A-1797773
2.3E-03	1.3E-06	2.4E-01	CD	SNP_A-1797773
2.1E-03	4.0E-07	9.9E-01	CD	SNP_A-1817967
3.0E-03	1.4E-05	1.9E-02	RA	SNP_A-1858955

Table S4: p **values of SNPs identified using the no-call-only filter.**

p_d	p_n	p_h	Disease	SNP
0.19	3.7E-10	0.09	BD	rs10238378
0.21	8.4E-10	1.00	BD	rs10485575
0.77	1.7E-14	0.56	RA	rs10768666
0.46	9.1E-11	0.52	BD	rs10811497
0.31	4.0E-14	0.23	BD	rs10896468
0.05	3.6E-10	0.91	CD	rs10896468
0.03	9.1E-10	0.80	T2D	rs10896468
0.14	3.0E-18	0.59	BD	rs11228904
0.07	1.2E-10	0.19	T1D	rs11228904
0.50	1.8E-10	0.91	HT	rs11228904
0.93	1.5E-09	1.00	HT	rs11583656
0.80	3.3E-14	0.86	BD	rs1191684
0.74	1.5E-09	0.68	BD	rs12428824
0.66	4.7E-10	0.40	T1D	rs1421867
0.35	7.1E-13	0.97	HT	rs17080801
0.17	3.9E-09	0.72	BD	rs17080801
0.35	2.4E-10	0.97	T2D	rs17310770
0.26	1.0E-12	0.02	HT	rs17423694
0.03	9.1E-11	0.57	BD	rs17636964
0.86	1.4E-10	0.97	T2D	rs17636964
0.31	2.7E-10	0.91	RA	rs17636964
0.49	1.2E-15	0.40	T1D	rs1809667
0.41	3.7E-12	0.93	HT	rs1819829
0.84	3.1E-10	0.27	RA	rs1820450
0.41	7.2E-10	0.17	BD	rs1868584
0.07	3.6E-11	0.36	T1D	rs1930171
0.57	3.6E-09	0.87	T2D	rs2039945
0.86	4.3E-10	0.88	HT	rs2804672
0.88	4.6E-14	0.75	BD	rs3864439
0.90	2.6E-14	0.87	RA	rs4236384
0.61	3.8E-09	0.79	T2D	rs4318932
0.47	2.6E-12	0.15	T2D	rs4471699
0.02	4.4E-10	0.83	BD	rs4471699
0.37	1.4E-09	0.11	CAD	rs4532803
0.16	6.3E-09	0.83	HT	rs4545817
0.02	1.6E-16	0.09	BD	rs584630
0.63	2.4E-09	0.52	RA	rs6494831
0.09	4.0E-16	0.06	RA	rs6510085
0.61	8.5E-09	0.51	CAD	rs6512631
0.12	8.9E-14	1.00	HT	rs7319991
0.06	2.6E-12	0.17	T1D	rs7319991
0.38	8.1E-12	0.28	BD	rs7319991
0.11	9.8E-10	1.00	T2D	rs7319991
0.14	1.8E-09	0.05	CAD	rs7319991
0.91	9.9E-10	0.95	T1D	rs8182488
0.75	1.1E-10	0.96	BD	rs9948005
0.40	8.5E-10	0.30	RA	rs9976299
0.19	3.0E-19	0.84	CAD	SNP_A-1817967
0.46	2.0E-11	0.77	BD	SNP_A-1858955
0.80	9.5E-10	0.47	CD	SNP_A-1858955

studied, consistent with the presence of several high-homology duplicons in the human reference sequence. The results of Alkan et al. are consistent with the present results, because even high homology sequences have differences. The presence of at least one well-defined cluster in the cluster plots, togther with an observation of HWE in the control populations, suggests that the SNP occurs in just one of the duplicons.

Stringent Filter Conditions

To clarify possible correlations between the stringent filter conditions, I analyze each condition alone and in pairwise combinations with other conditions. I use the 90%, 1000bp homology criterion rather than the 85%, 300bp condition to allow an automated analysis. The set of SNPs analyzed for a population is the subset (a) that is not excluded by the WTCCC analysis [11], and (b) for which the SNP has more than 10 individuals within the control population for each of the AA/AB/BB genotypes. There are 344,344 such SNPs.

The results are summarized in Table S6 for SNPs without homology, and Table S7 for SNPs with homology. The DIST condition refers to the Chi-squared test for a common distribution. From the numbers in Tables S6 and S7, the following observations can be made for all populations:

- Among SNPs meeting the DIST condition, there is a roughly tenfold higher probability (∼10%) of a no-call than for SNPs not meeting the DIST condition (∼1%). Conversely, among SNPs meeting the no-call condition, there is a roughly tenfold higher probability (∼1%) of meeting the DIST condition than for SNPs not meeting the no-call condition (∼0.1%).
- Among SNPs meeting the HWE condition, there is a roughly tenfold higher probability (∼10%) of a no-call than for SNPs not meeting the HWE condition (∼1%). Conversely, among SNPs meeting the no-call condition, there is a roughly tenfold higher probability (∼1%) of meeting the HWE condition than for SNPs not meeting the no-call condition (∼0.1%).
- Among SNPs in homologous regions, there is a roughly 2.5-fold increase in the probability (∼0.25%) of meeting the HWE condition compared with SNPs not in homologous regions (∼0.1%). Conversely, among SNPs meeting the HWE condition, there is a roughly 2.5-fold increase in the probability (∼3.3%) of being in a homologous region compared to SNPs not meeting the HWE condition (∼1.3%).

The first two interactions are due to the clustering algorithm. When clusters are nearby, the intermediate points are likely to be no-calls. The bias induced by the grouping of no-calls can appear to generate

Table S5: **Copy Number Variation at Stringent Filter Loci.**

SNP	Known CNV Data from TCAG
rs4471699	13 of 270 HapMap individuals show CNV in a 300kb region including this SNP [201].
rs669980	8 of 30 HapMap samples show a gain at a 260kb region including this SNP [202].
rs10502407	—
rs12134625	—
rs12381130	3 of 270 HapMap individuals show CNV in a 450kb region including this SNP [201].
rs935019	—
rs11060028	—
rs9551988	2 of 2,026 healthy controls show a gain in a 350kb region including this SNP [203].
rs3805006	—
rs295470	—
rs12227938	6 of 270 HapMap individuals show CNV in a 1,200kb region including this SNP [201].
SNP_A-1797773	This SNP maps to two genomic regions on chromosome 16. The region at 34Mb has 2 out of 50 healthy controls show a gain in a 470kb region including this SNP [204].
rs9378249	20 of 270 HapMap individuals show CNV in an 84kb region including this SNP [201].
rs12070036	—
rs11010908	—
rs4988327	—
rs841245	—
rs2122231	—
rs9839841	—
SNP_A-1948953	—
rs4850057	20 of 126 / 36 of 270 HapMap individuals show a loss in an 11kb region including this SNP [205, 206]. 146 of 2,026 healthy controls show a loss in a 9kb region including this SNP [203].

CNV loci from TCAG are shown when a single cited study has identified multiple individuals with variation at a locus.

Table S6: **Interdependence of the stringent filter criteria for SNPs without 90% homology.**

No-call	DIST	HWE	BD	CD	CAD	HT	RA	T1D	T2D
0	0	0	334871	338468	337968	336612	337138	336418	336746
1	0	0	4117	521	1045	2452	1681	1950	2331
0	1	0	330	486	283	331	540	952	292
0	0	1	285	196	313	234	264	247	262
1	1	0	24	2	7	23	21	16	10
0	1	1	35	17	35	21	33	94	37
1	0	1	18	2	34	17	9	10	8
1	1	1	18	6	13	8	12	11	12

The No-call, DIST, and HWE columns indicate the conditions used in the stringent filter. A value of 1 indicates that the condition is true, and a value of 0 indicates that the condition is false.

Table S7: **Interdependence of the stringent filter criteria for SNPs with at least 90% homology.**

No-call	DIST	HWE	BD	CD	CAD	HT	RA	T1D	T2D
0	0	0	4554	4617	4606	4593	4582	4566	4586
1	0	0	73	14	21	43	32	28	44
0	1	0	7	5	4	2	23	36	3
0	0	1	10	6	10	2	5	9	8
1	1	0	1	1	0	0	1	1	1
0	1	1	0	2	3	2	2	5	2
1	0	1	1	0	1	2	0	1	2
1	1	1	0	1	1	2	1	0	0

The No-call, DIST, and HWE columns indicate the conditions used in the stringent filter. A value of 1 indicates that the condition is true, and a value of 0 indicates that the condition is false.

changes in called genotype distribution, including changes in HWE.
The third interaction is likely to be due to the influence of SNPs with homologs containing identical flanking sequence to the SNP. Such SNPs will have cluster plots in which the clusters are closer together, leading to higher no-call rates and apparent HWE violations.
The three stringent test conditions are correlated, but the magnitude of the correlation is small relative to the selectivity of each condition. Thus, there is meaningful complementary information in each of the conditions.
Additional information can be observed for particular populations. For T1D and RA, SNPs have a threefold higher rate of meeting the DIST condition when the SNP is in a homologous region compared to SNPs not in homologous regions. Conversely, a SNP has a threefold higher rate of being in a homologous region if it meets the DIST condition compared to when it does not. A closer inspection of the data reveals that the qualifying SNPs are concentrated in the MHC region of chromosome 6, a region that is known to be associated with both T1D and RA in this dataset [11].
The no-call condition used by the stringent filter has a p value of 5×10^{-5}. Figure S2 shows the probability that a SNP will meet this condition for each population. Many more SNPs qualify than would be expected for such a small p value. Nevertheless, the present analysis only uses the p value as a threshold to generate candidate SNPs, and does not rely on the precision of this statistic. Figure S2 also shows that the no-call behavior is not uniform across populations. The rate for BD is almost tenfold higher than the rate for CD. Such variation between populations could represent a disease-related phenomenon, but the explanation for the variation remains unclear.

SNP Interactions

When multiple SNPs are associated with a disease, there could be an interaction in which somatic mutations in two SNPs occur more frequently than would be expected if they were independent risk factors. An interaction analysis will also identify whether or not there is a small number of individuals who tend to generate intermediate points in multiple cluster plots, explaining most of the results. (For example, there was a small number of individuals in the WTCCC data who generated outlying low-intensity points at multiple loci in the CAD/RA/NBS cohorts, a probable artifact of different procedures for those cohorts [11].)
To analyze interaction, each of the associations identified by the stringent filter is processed to identify the individuals who received a no-call at that locus. The no-call is a proxy for a significant amount of gene

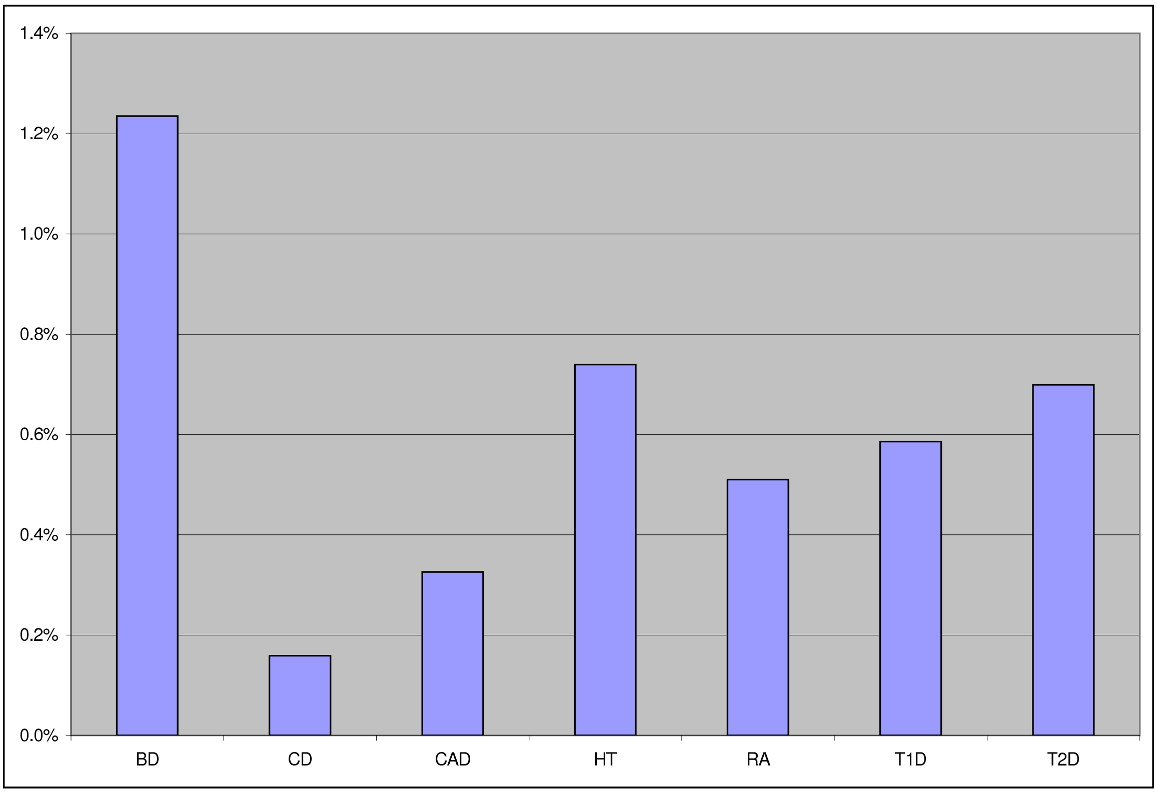

Figure S2: **Probability of a SNP meeting the no-call condition for each population.**

conversion. Individuals who received no-calls at more than 5% of the SNPs were excluded, since correlated experimental error across all SNPs could lead to spurious indicators of SNP interaction. (At most 2.2% of individuals were excluded in this way from any population.)

To increase the data set size, stringent-filter SNPs are included for diseases in which there is a relaxed-filter association. Due to effects like those illustrated in Figure S1, the seven SNPs showing cluster boundary shifts are excluded from the interaction analysis. There is no interaction data for CD or RA because fewer than two SNPs remain after this exclusion. The results for the remaining five diseases are shown in Tables S10 through S12.

In each table, the expected number of individuals with no-calls at both loci is computed using the marginal no-call distributions assuming independence. The actual number is shown, and the p value for a 2x2 chi-squared test of independence is given.

For most of the SNP pairs, there is no significant interaction. For a small number of SNPs, there does seem to be a higher than expected frequency of joint no-calls. The set of SNPs { rs10502407, SNP_A-1797773,

Table S8: **SNP interactions for BD.**

SNP_1	no-calls	SNP_2	no-calls	Actual	Expected	p
rs10502407	115	SNP_A-1797773	95	21	5.5	3.9E-12
rs12070036	100	SNP_A-1797773	95	18	4.8	2.6E-10
rs10502407	115	rs12070036	100	16	5.8	8.4E-06
rs10502407	115	rs9551988	90	14	5.2	5.5E-05
rs12070036	100	rs9551988	90	8	4.6	0.09
rs2122231	136	rs9551988	90	4	6.2	0.35
rs12227938	106	SNP_A-1797773	95	7	5.1	0.38
rs12227938	106	rs9551988	90	3	4.8	0.38
rs4850057	89	SNP_A-1797773	95	6	4.3	0.38
rs12227938	106	rs4850057	89	3	4.8	0.39
rs2122231	136	rs4850057	89	8	6.1	0.42
rs10502407	115	rs12227938	106	8	6.2	0.44
rs12070036	100	rs4850057	89	3	4.5	0.45
rs4850057	89	rs9551988	90	5	4.1	0.62
rs12070036	100	rs2122231	136	6	6.9	0.72
rs9551988	90	SNP_A-1797773	95	5	4.3	0.74
rs2122231	136	SNP_A-1797773	95	6	6.5	0.82
rs12070036	100	rs12227938	106	5	5.4	0.87
rs12227938	106	rs2122231	136	7	7.3	0.90
rs10502407	115	rs4850057	89	5	5.2	0.93
rs10502407	115	rs2122231	136	8	7.9	0.98

Population is 1973 after excluding individuals with more than 5% no-calls.

and rs12070036, rs9551988, rs12134625, rs295470, rs389600, rs9257223, rs12381130, rs11010908 } has the property that each SNP in the set is positively correlated with another member in at least one population with $p < 0.005$. Of the ten members of this set, seven show evidence of a difference between the 58C and NBS cluster plots (Table 9).

Looking at the cluster plots for these SNPs. the no-calls are concentrated at the low-intensity region of the clusters, closest to the origin. There is likely to be some correlation between intensity at different loci simply due to experimental variation in total quantity of DNA being analyzed for each individual. This correlation could explain the observed interaction effects. Supporting this explanation (and arguing against a disease-associated interaction), there is an increased joint frequency of these loci even in the NBS population (Table S13).

Nevertheless, the correlated intensity phenomenon accounts for a relatively small fraction of the data for an interacting SNP pair. No more than 21% of the no-calls for a locus are for individuals with no-calls at any other locus.

Table S9: **SNP interactions for CAD.**

SNP_1	no-calls	SNP_2	no-calls	Actual	Expected	p
rs12134625	118	rs9551988	91	17	5.5	1.9E-07
rs295470	159	rs9551988	91	15	7.4	2.6E-03
rs12134625	118	rs12227938	101	11	6.0	0.03
rs10502407	126	rs295470	159	15	10.2	0.10
rs12227938	101	rs295470	159	4	8.2	0.12
rs10502407	126	rs12227938	101	3	6.5	0.15
rs12227938	101	rs9551988	91	7	4.7	0.26
rs10502407	126	rs9551988	91	8	5.8	0.34
rs10502407	126	rs12134625	118	9	7.6	0.58
rs12134625	118	rs295470	159	10	9.5	0.87

Population is 1965 after excluding individuals with more than 5% no-calls.

Table S10: **SNP interactions for HT.**

SNP_1	no-calls	SNP_2	no-calls	Actual	Expected	p
rs841245	160	rs935019	101	15	8.2	0.01
rs2122231	160	rs9551988	118	3	9.5	0.02
rs841245	160	rs9551988	118	16	9.5	0.02
rs12227938	123	rs935019	101	2	6.3	0.07
rs2122231	160	rs935019	101	4	8.2	0.12
rs12227938	123	rs841245	160	6	9.9	0.18
rs12227938	123	rs2122231	160	13	9.9	0.30
rs2122231	160	rs841245	160	10	12.9	0.37
rs935019	101	rs9551988	118	8	6.0	0.39
rs12227938	123	rs9551988	118	9	7.3	0.51

Population is 1978 after excluding individuals with more than 5% no-calls.

Table S11: **SNP interactions for T1D.**

SNP_1	no-calls	SNP_2	no-calls	Actual	Expected	p
rs389600	91	rs9257223	127	16	5.8	7.5E-06
rs12381130	89	rs9257223	127	15	5.7	3.5E-05
rs12227938	101	rs12381130	89	1	4.5	0.08
rs12381130	89	rs389600	91	2	4.1	0.28
rs12227938	101	rs9257223	127	8	6.4	0.51
rs12227938	101	rs389600	91	5	4.6	0.85

Population is 1992 after excluding individuals with more than 5% no-calls.

Table S12: **SNP interactions for T2D.**

SNP_1	no-calls	SNP_2	no-calls	Actual	Expected	p
rs10502407	118	SNP_A-1797773	93	20	5.6	1.1E-10
rs11010908	132	SNP_A-1797773	93	17	6.2	4.8E-06
rs10502407	118	rs9551988	85	14	5.1	3.2E-05
rs11010908	132	rs10502407	118	17	7.9	5.6E-04
rs4850057	95	rs9551988	85	8	4.1	0.04
rs9551988	85	SNP_A-1797773	93	6	4.0	0.30
rs11010908	132	rs9551988	85	7	5.7	0.56
rs11010908	132	rs4850057	95	7	6.4	0.79
rs4850057	95	SNP_A-1797773	93	4	4.5	0.81
rs10502407	118	rs4850057	95	6	5.7	0.89

Population is 1969 after excluding individuals with more than 5% no-calls.

Table S13: **SNP interactions for NBS.**

SNP_1	no-calls	SNP_2	no-calls	Actual	Expected	p
rs10502407	96	rs12070036	48	8	2.3	3.2E-03
rs12070036	48	SNP_A-1797773	34	4	0.8	4.1E-03
rs12070036	48	rs9551988	48	3	1.2	0.22
rs10502407	96	rs9551988	48	5	2.3	0.25
rs10502407	96	SNP_A-1797773	34	2	1.6	0.90
rs9551988	48	SNP_A-1797773	34	1	0.8	0.93

Population is 1498 after excluding individuals with more than 5% no-calls.

Table S14: **Duplicons having identity lower than the stringent filter threshold of 85%, but otherwise meeting the stringent filter conditions.**

SNP	Chromosome	Disease	Identity	Duplicon length	Characterized genes in duplicons
rs9353332	6	HT	83%	1.9kb	—
rs2073149	6	T1D	82%	1.7kb	OR12D2
rs4370913	11	CAD	81%	0.6kb	—
rs4367490	8	CAD	81%	2.4kb	PDGFRL
rs1464336	4	RA	80%	0.4kb	—
rs2683780	3	BD	74%–79%	0.9–2.4kb	—
rs11173071	12	CAD	78%	3.7kb	—
rs228068	21	CAD	77%	0.4kb	SLC37A1
rs1410707	9	RA	73%–76%	0.4–0.6kb	ZPLD1, KHDRBS2
rs7247513	19	BD	74%–75%	1kb–1.8kb	ZNF490, ZNF14, ZNF709
rs887622	7	BD	74%	0.5kb	CREB5
rs10917688	1	BD	71%	0.6kb	—

Duplicons with Lower Identity

Eleven SNPs with identity between duplicons of 71%-83% were identified. These SNPs are summarized in Table S14. The degree of identity across the duplicon is lower than would be expected for gene conversion [1]. Nevertheless, it is possible that nonuniform identity within the duplicon creates opportunities for conversion, as illustrated previously for IDS [3]. For example, in a 60bp region surrounding the SNP rs887622, there is 97% identity.

Mock Association Study

I identified ten SNPs for each disease, chosen to reside on known segmental duplications from the segmental duplication database. A chi-squared statistic comparing the distributions of called genotypes in controls and in the disease samples was computed, and the ten SNPs that minimized this statistic were chosen. (The selected SNPs for a disease sample are therefore those whose genotype distributions are closest to the controls.) For each disease I searched for disease associations using the literature in the same way that associations were sought for SNPs selected by the various filters. The details are presented in Tables S15 and S16.

Table S15: **Table of "associations" for the mock study, part 1.**

Disease	SNP	Chr	Characterized Genes in Duplicons	Evidence-level [citations]	Chi-square value
CAD	rs17420195	1	NBPF3, NBPF20, NBPF7, NBPF1, NBPF10, NBPF8, NBPF14, NBPF20, NOTCH2NL, [ALPL], [REG4]	0	0.006042
HT	rs11207936	1	L1TD1, FGF14	0	0.003922
T1D	rs12463570	2	IGKV3-15, RMND5A	6 for IGKV3-15 [207]	0.00155
RA	rs2741029	2	UGT1A8, UGT1A10	0	0.003265
CAD	rs7424996	2	PDCD6, [SDHALP1], [AHRR], [SDHAP3], [SDHALP2]	6 for PDCD6 [208]	0.001202
HT	rs7635788	3	—	0	0.003433
RA	rs9829609	3	CICE, [FANCD2], [TMEM111]	0	0.000846
BD	rs6832554	4	KHDRBS3, MAPRE1, [EFCAB8]	1 for MAPRE1 [209]	0.00003
HT	rs9999103	4	UGT2B28, [UGT2B11]	3 for UGT2B28 (androgen/estrogen metabolism) [210]	0.001416
RA	rs2867698	4	[ANTXR2]	0	0.00384
CD	rs2202039	4	[FAM27C], [FAM27E3], [FAM27A], [FAM27B]	0	0.000768
T1D	rs1910787	5	RUFY3	0	0.0007
BD	rs1592792	5	THOC3, FAM153B, FAM153A	0	0.003115
CD	rs13219662	6	—	0	0.000275
T2D	rs574710	6	—	0	0.000999
CAD	rs6952677	7	—	0	0.004498
CAD	rs12539799	7	FAM182A	0	0.000545
HT	rs2706984	7	—	0	0.001838
T2D	rs6949430	7	ZNF735, ZNF479, ZNF716, ZNF679	0	0.000932
T2D	rs2164110	7	ZNF679	0	0.000398
CD	rs6974327	7	[GPC3]	0	0.004082
T1D	rs11770635	7	—	0	0.002326
RA	rs2373680	7	ACTR3B	0	0.003545
CD	rs6962199	7	ACTR3B	0	0.004236
T2D	rs17656755	8	[DEFA5], [TRPC2], [ASNS]	0	0.002557
BD	rs13268588	8	LRRC69, CP-pseudogene, HPS3	0	0.000303
RA	rs1838182	8	TMEM41B, CEP164, [BACE1], [FCGBP], [PSMC4]	6 for FCGBP [211]	0.003583
T1D	rs10975106	9	RNF152	0	0.000708
CAD	rs1337577	9	PRSS3, TMEM45B	0	0.0054
T1D	rs7910625	10	[PDSS1]	0	0.001389
T2D	rs16930315	10	SVIL	0	0.000581
CD	rs2754428	10	PLD5	0	0.000201
T1D	rs10900177	10	—	0	0.00233
T2D	rs2801023	10	—	0	0.002706
CAD	rs12411806	10	EIF5AL1, FAM22A, [ANXA11]	2 for ANXA11 [212]	0.006606
BD	rs4052539	11	MRGPRX3, [SAA3], [SAAL1], [GRK5], [PRDX3], [SFXN4]	4 for PRDX3 [213, 214]	0.001576
CAD	rs7102961	11	SEPT13, SEPT7, CDC10L	3 for SEPT7 (interaction with S100A4/MTS1) [92–95, 215]	0.004201

Genes in square brackets are outside the duplicons, but a duplicon is at most 30kb upstream of the gene.

Table S16: **Table of “associations” for the mock study, part 2.**

Disease	SNP	Chr	Characterized Genes in Duplicons	Evidence-level [citations]	Chi-square value
T1D	rs4080494	11	—	0	0.000943
CD	rs10770298	12	ZNF705A, ZNF705D, [FAM66C], [FAM66D], [FAM66A], [FAM66E], [DEFB108B]	0	0.000449
RA	rs7314384	12	KLRC1, KLRC2, KLRC3, [KLRC4], [KLRK1]	6 for KLRC1 and KLRC2 [216]	0.001736
BD	rs10842851	12	PPFIBP1	0	0.00238
T1D	rs10771818	12	[OVOS2]	0	0.003173
HT	rs4964963	12	—	0	0.002852
HT	rs9506650	13	—	0	0.000842
RA	rs11619689	13	[PARP4]	1 for PARP4 [152]	0.000196
CAD	rs9561068	13	GPC5	0	0.000897
CAD	rs7496817	15	PWRN1, [PWRN2]	0	0.004715
T1D	rs17841165	15	WHAMM, WHAMML1, WHAMML2, [FSD2]	0	0.002965
CD	rs7496269	15	—	0	0.003858
CAD	rs2908784	16	GOLGA8G, [HERC2], [HERC2P2], [HERC2P3], [GOLGA8E]	0	0.005395
HT	rs7221571	17	[ANKRD30B]	0	0.003198
HT	rs11080053	17	—	0	0.003701
CD	rs4795333	17	ARL17, LRRC37A, [LRRC37A4]	0	0.004252
HT	rs4795333	17	ARL17, LRRC37A, [LRRC37A4]	0	0.003241
RA	rs11080434	18	[CIDEA]	0	0.002597
RA	rs11080786	18	[ANKRD30B]	0	0.001555
RA	rs9963735	18	[ANKRD30A], [ANKRD30B]	0	0.001381
BD	rs1919833	18	—	0	0.000915
BD	rs1044409	19	FEM1A	0	0.004161
BD	rs12710122	19	ZNF66, ZNF737	0	0.003255
T2D	rs12985617	19	ZNF100	0	0.004257
CD	rs4933027	19	—	0	0.000254
T1D	rs2862789	19	ZNF181, ZNF302	0	0.00147
CD	rs678812	19	KIR3DP1, KIR2DS2, KIR2DL3, KIR2DL2, KIR2DL4, KIR3DL1, KIR103, KIR2DS4, KIR3DL2, KIR2DS3, KIR2DL5A, KIR2DS5, KIR2DS1, KIR3DP1, KIR3DS1, KIR2DL5B, [FCAR]	3 (NK cell receptors; NK cell activity reduced in CD) [217]	0.000168
BD	rs13044242	20	CST1, CST2, CST4, [CST3]	0	0.000744
T2D	rs7410107	21	ANKRD30B	0	0.004221
T2D	rs11088226	21	—	0	0.002704
BD	rs11089263	22	CCT8L2, psiTPTE22, CCT8L1, FABP5L3, MLL3	0	0.003991
HT	rs9306387	22	IGLL3, F[gamma]8	0	0.000723
T2D	rs3912046	22	CRYBB2	0	0.002856

Genes in square brackets are outside the duplicons, but a duplicon is at most 30kb upstream of the gene.
